# Supplementary material for: Analysis of extracellular mRNA in human urine reveals splice variant biomarkers of muscular dystrophies
Source: Nat Commun. 2018 Sep 25;9:3906. doi: 10.1038/s41467-018-06206-0 (PMC6156576; doi:10.1038/s41467-018-06206-0)
Supplement: Supplementary file 1 — Supplementary Information [file 41467_2018_6206_MOESM1_ESM.pdf]

Analysis of extracellular mRNA in human urine reveals splice variant biomarkers of muscular dystrophies

Layal Antoury[1,4], Ningyan Hu[1,4], Leonora Balaj[1,4], Sudeshna Das[1,4], Sofia Georghiou[3,4], Basil Darras[3,4], Tim Clark[1,4], Xandra O. Breakefield[1,2,4], and Thurman M. Wheeler[1,4,*]

[1]Departments of Neurology and [2]Radiology, Massachusetts General Hospital, [3]Department of Neurology, Boston Children's Hospital, and [4]Harvard Medical School, Boston, MA

[*]Corresponding author:

twheeler1@mgh.harvard.edu

Contents:

Supplementary Figures 1 - 18

Supplementary Tables 1 - 11

Supplementary Discussion

Supplementary References

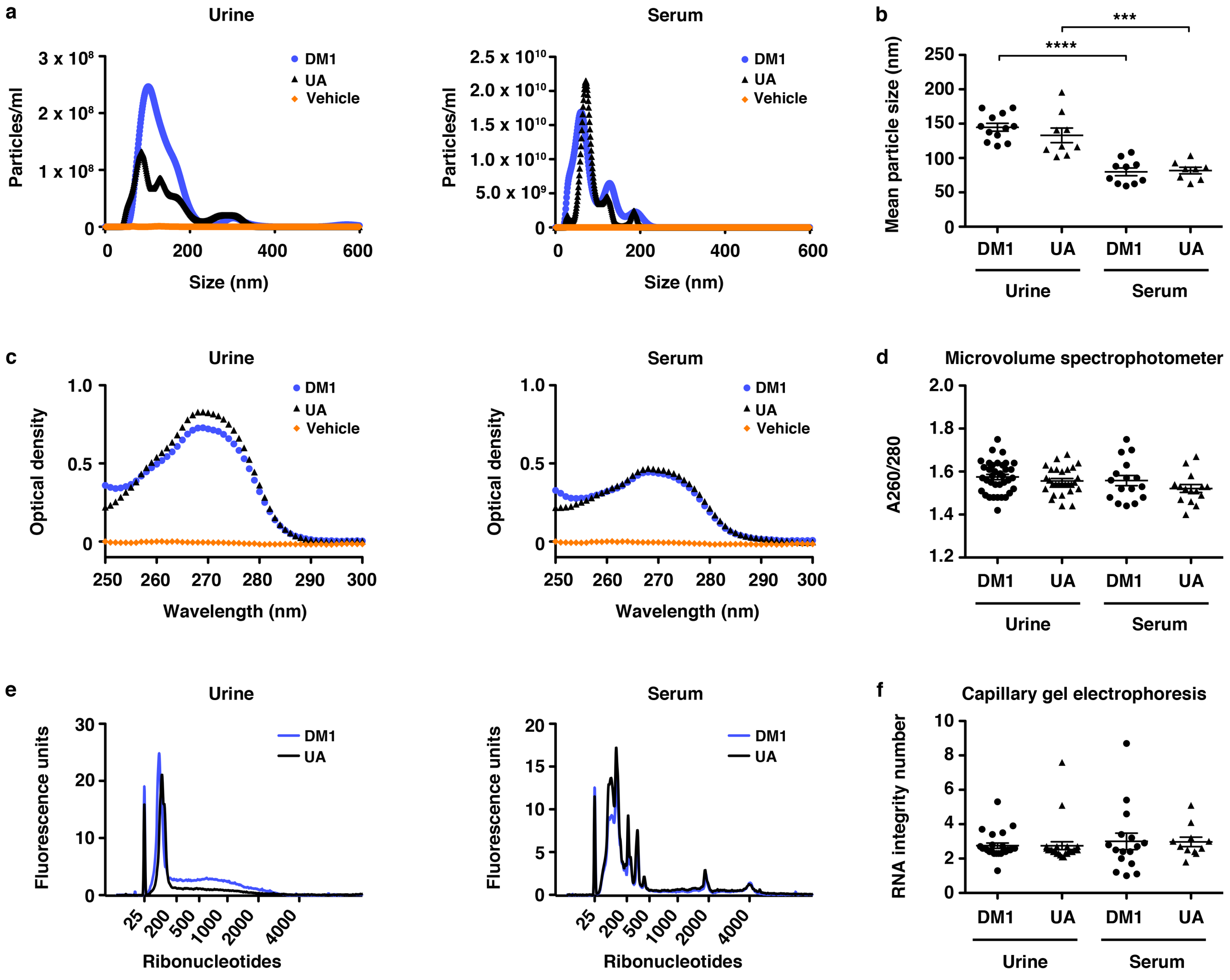

Supplementary Figure 1 Characterization of exRNA in human urine and serum from DM1 and unaffected (UA) control subjects. (**a**) We used particle imaging and tracking analysis software to measure extracellular particle size and concentration in urine and serum samples from DM1 (N = 12 urine, 10 serum) and UA control (N = 9 urine, 8 serum) subjects. Representative traces of particle size from DM1 and UA subjects in urine (left) and serum (right) are shown. Note that the tracing for vehicle (saline) overlies the x axes. (**b**) Mean particle size from each individual sample in urine (left) and serum (right). **** $P < 0.0001$; *** mean difference 51.2, 95% CI of difference 21.9 to 80.6; one-way ANOVA. (**c**) Optical density spectra of exRNA from urine (N = 36 DM1, 28 UA) and serum (N = 23 DM1, 19 UA) samples using a microvolume spectrophotometer (Nanodrop). Vehicle (water) served as reference. The peak of 270 nm reflects an artifact of residual Trizol that was used to purify the RNA[1]. (**d**) Optical density values of urine and serum exRNA calculated as the ratio of absorbance at 260 nm/absorbance at 280 nm. (**e**) We used capillary gel electrophoresis (Agilent Bioanalyzer) to analyze the concentration, size distribution, and quality of exRNA in urine (N = 36 DM1, 28 UA controls) and serum (N = 16 DM1, 14 UA controls). Representative electropherogram traces of exRNA size in nucleotides (nt) from urine (left) and serum (right). (**f**) RNA integrity number (RIN) in urine (left) and serum (right), as calculated from electropherogram traces using a software algorithm (Agilent). All error bars indicate mean ± s.e.m.

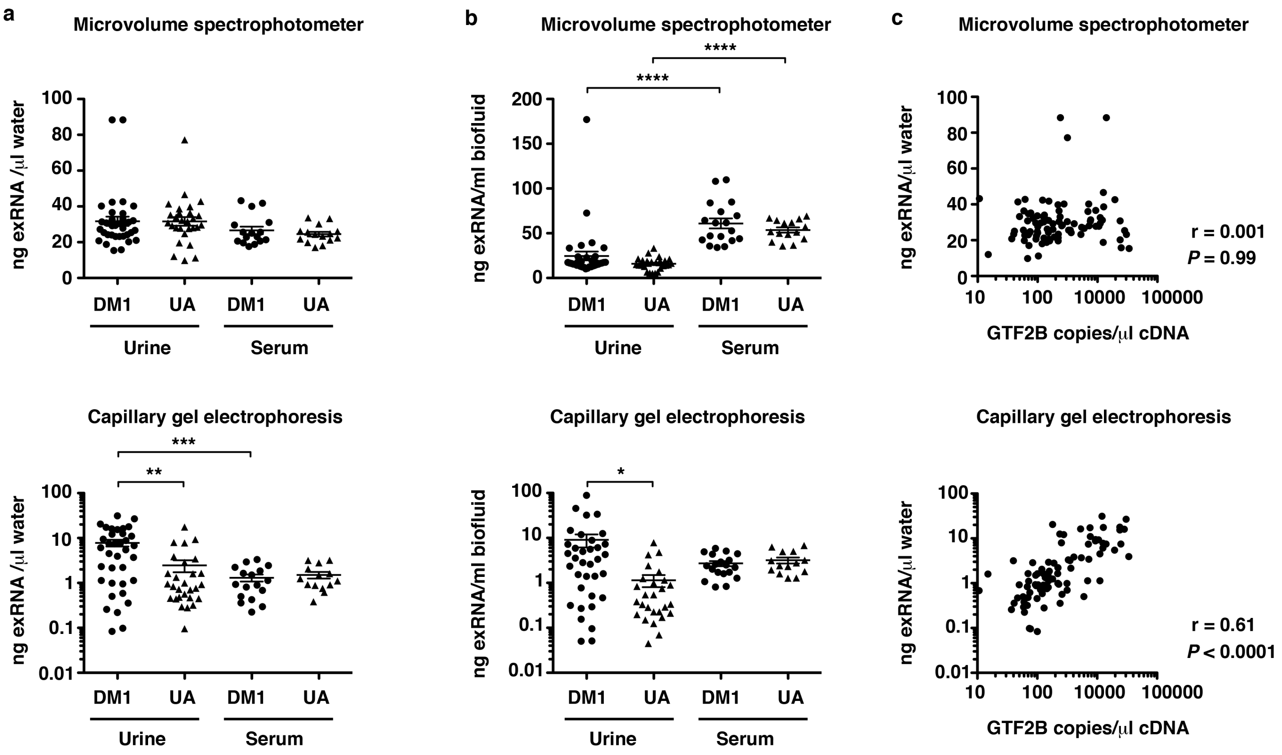

Supplementary Figure 2 Comparison of methods to estimate exRNA concentration and yield from urine and serum. We examined exRNA from urine (N = 36 DM1, 28 UA controls) and serum (N = 16 DM1, 14 UA) using both microvolume spectrophotometry and capillary gel electrophoresis (see Supplementary Fig. 1). (**a**) Estimations of purified exRNA concentration in RNase-free water using optical density values at 260 nm measured by a microvolume spectrophotometer (upper) and fluorescence intensity measured by capillary gel electrophoresis (lower). Note that the estimation of exRNA concentration measured by a microvolume spectrophotometer was several-fold higher for DM1 urine, and an order of magnitude higher for UA urine and in serum of both groups, as compared to estimations of exRNA concentrations of these samples measured by capillary gel electrophoresis. *** mean difference 6.5, 95 CI of difference 2.2 to 10.8, DM1 urine vs. DM1 serum; ** mean difference 5.4, 95% CI of difference 1.7 to 9.0, DM1 urine vs. UA urine; *P* value < 0.0001, overall difference of means for all groups; one-way ANOVA. (**b**) Using concentration estimations in (**a**), we estimated the quantity of exRNA yield per volume of urine (20 - 120 ml; left) or serum (5.5- 8.5 ml; right) obtained from each participant. Individual data points from each sample are shown. **** *P* < 0.0001; * *P* = 0.02; one-way ANOVA. (**c**) Correlation of *General transcription factor 2B* (*GTF2B*) expression by droplet digital PCR (ddPCR), measured as copies per microliter cDNA, with the concentration of purified exRNA ng per microliter RNase-free water that was used to make the cDNA for ddPCR, as estimated by a microvolume spectrophotometer (upper) and capillary gel electrophoresis (lower). The correlation coefficient r and *P* value for each are shown. All error bars indicate mean ± s.e.m.

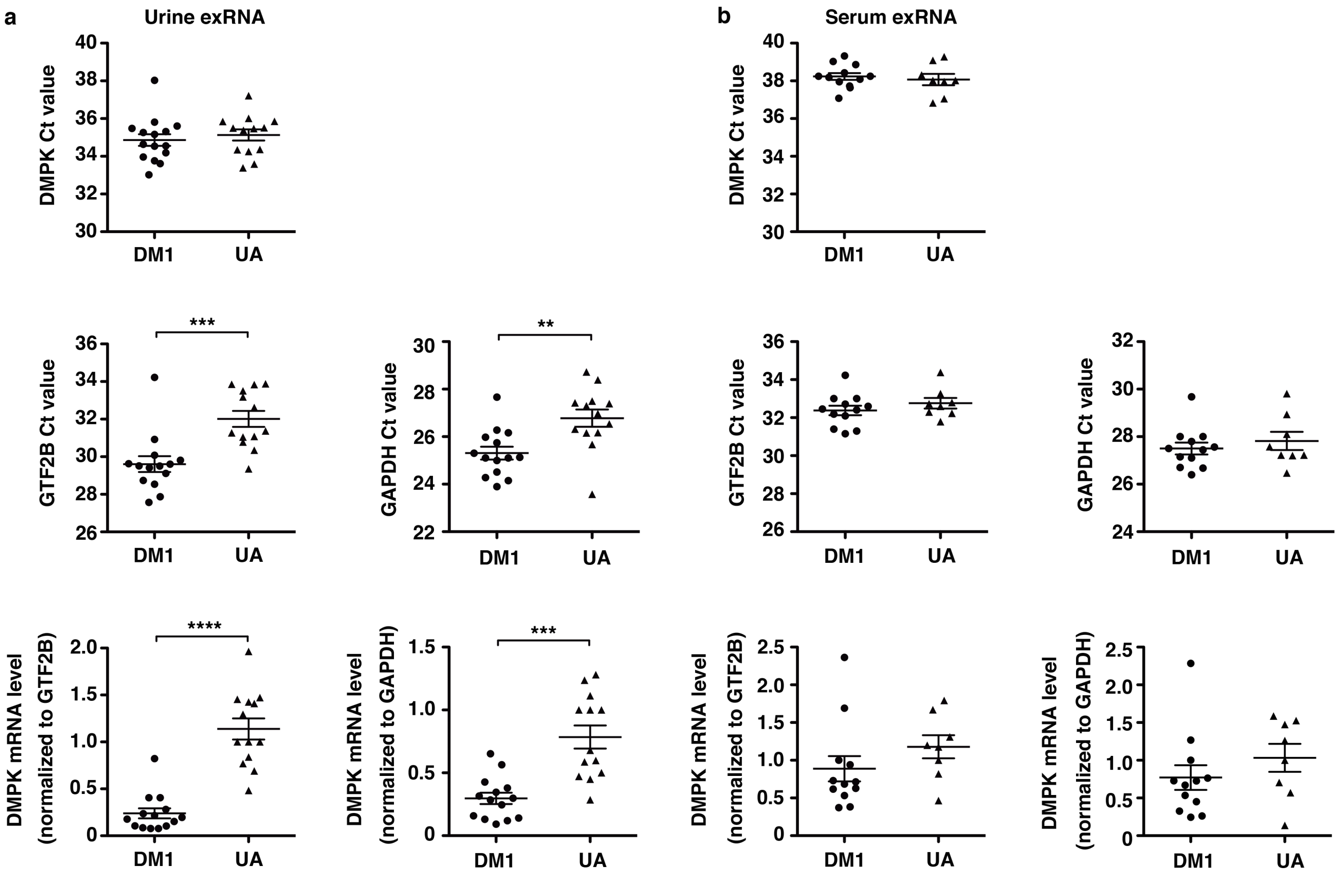

Supplementary Figure 3 *DMPK* gene expression in exRNA from urine and serum. We used Taqman qPCR to measure expression of *DMPK* and two reference genes, *General Transcription Factor 2B* (*GTF2B*) and *Glyceraldehyde Phosphate Dehydrogenase* (*GAPDH*), in urine and serum exRNA samples. (**a**) Upper and middle, expression of *DMPK*, *GTF2B*, and *GAPDH* expressed as cycle threshold (Ct) values (lower Ct values = higher expression) in urine exRNA from DM1 (N = 14) and UA (N = 13) subjects. Lower, *DMPK* expression normalized to *GTF2B* and *GAPDH* in urine exRNA. **** $P < 0.0001$ (DM1 vs. UA, *DMPK* normalized to *GTF2B*); *** $P = 0.0005$ (DM1 vs. UA, *GTF2B* Ct values), and $P = 0.0002$ (DM1 vs. UA, *DMPK* normalized to *GAPDH*); ** $P = 0.003$, (DM1 vs. UA, *GAPDH* Ct values); two-tailed *t* tests. (**b**) Upper and middle, expression of *DMPK*, *GTF2B*, and *GAPDH* expressed as Ct values in serum exRNA from DM1 (N = 12) and UA (N = 8) subjects. Lower, *DMPK* expression normalized to *GTF2B* and *GAPDH* in serum exRNA. All error bars indicate mean ± s.e.m.

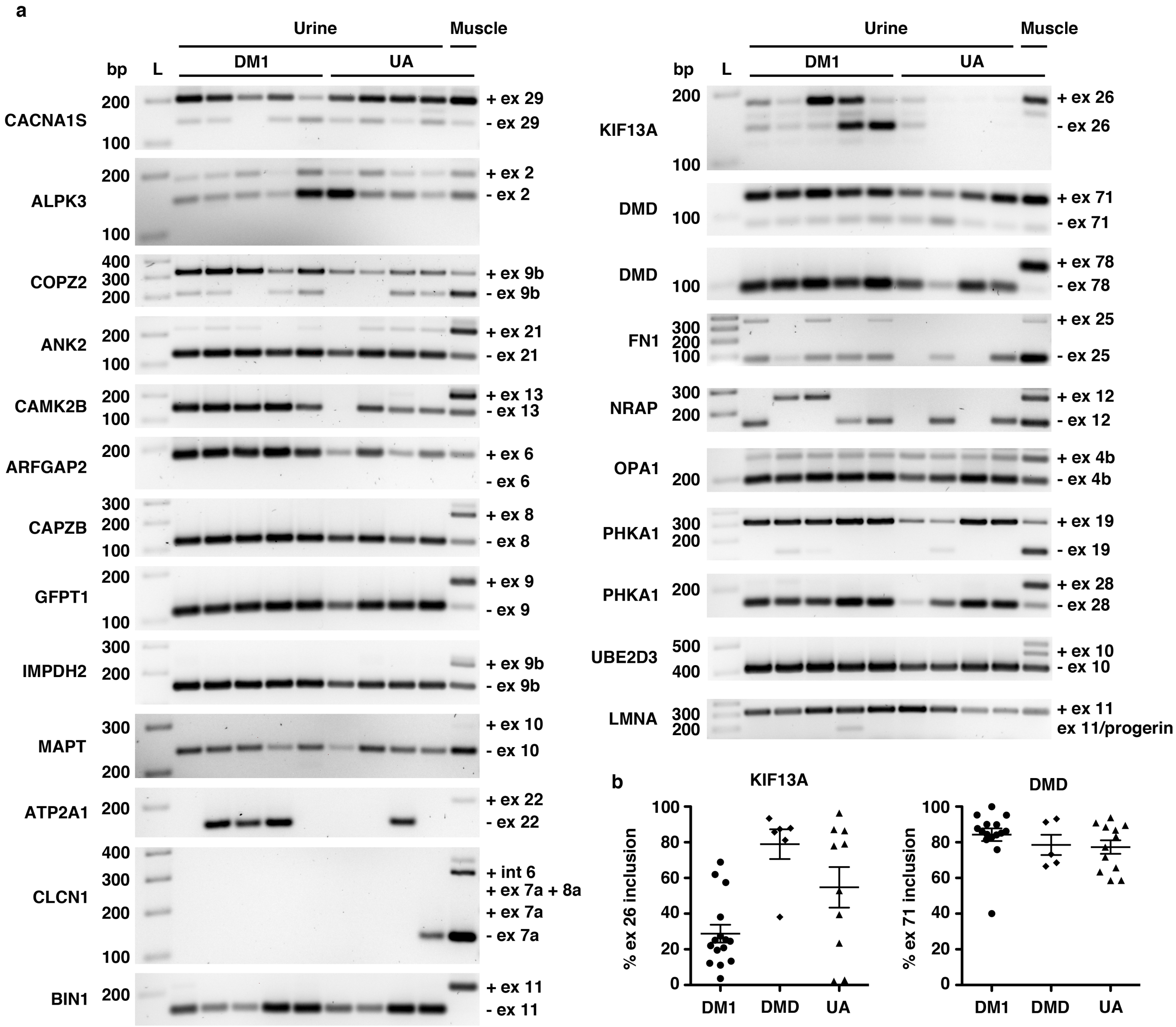

Supplementary Figure 4 Alternative splicing in urine exRNA isolated from DM1 and UA subjects. (**a**) We used RT-PCR to screen 23 candidate DM mis-regulated splice events[2] in urine exRNA from DM1 (N = 5) and UA control (N = 4) subjects. Splicing in normal muscle tissue served as a control. Transcript name is shown on the left and target exon/intron on the right. Splicing of *CAMK2B*, *ARFGAP2*, and *CAPZB* in an additional 5 DM1 and 3 UA control samples appeared identical to the samples shown above. "L" = DNA ladder. "bp" = base pairs. (**b**) Quantification of alternative splicing by RT-PCR of *KIF13A* exon 26 and *DMD* exon 71 in DM1 (N = 14), DMD/BMD (N = 6 or 5), and UA (N = 14 or 12) subjects. Note that *KIF13A* bands were absent in 1 DM1 and 4 UA subjects. Error bars indicate mean ± s.e.m.

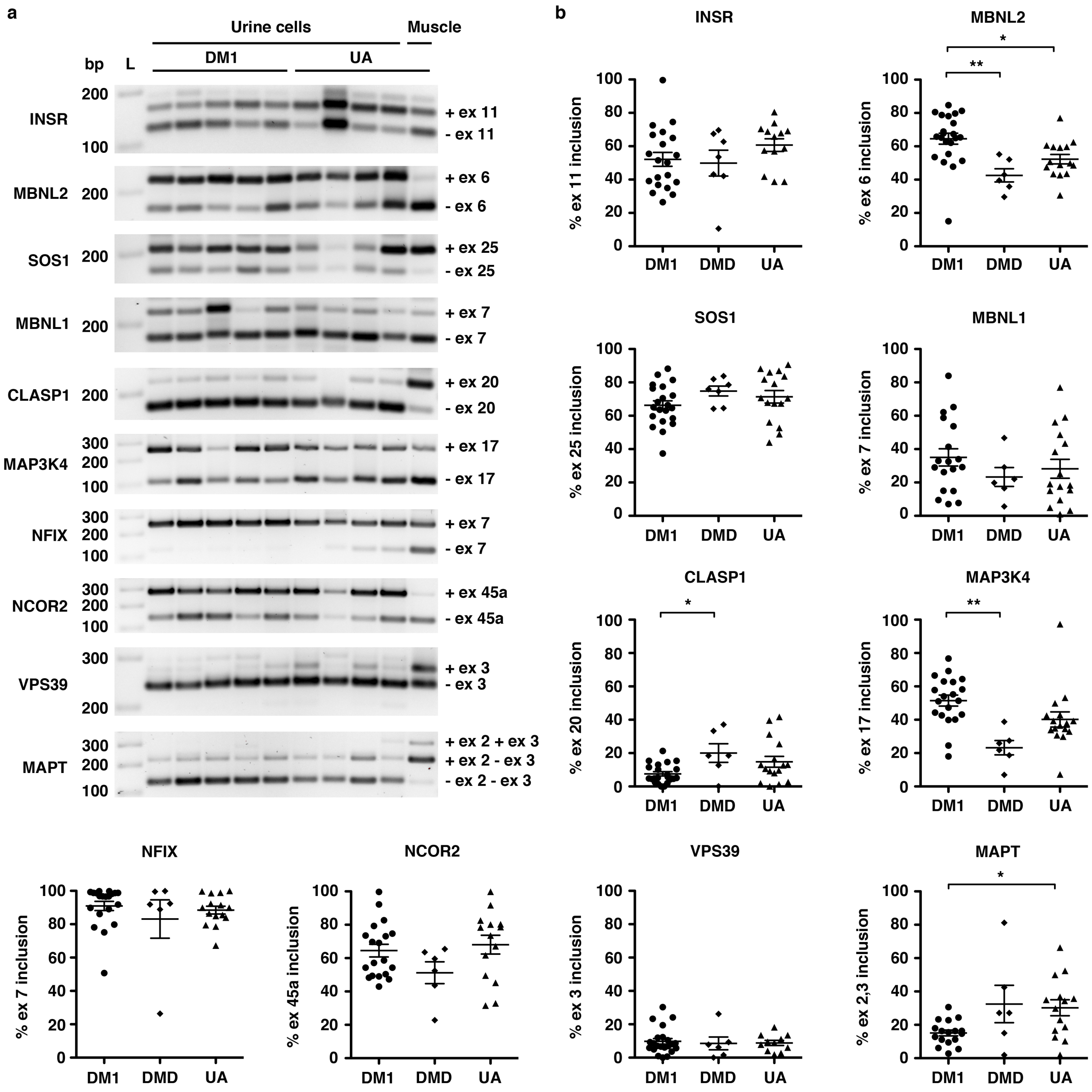

Supplementary Figure 5 Alternative splicing in total RNA from urine cells of DM1 and UA subjects. We isolated total RNA from urine cells of DM1 (N = 21), DMD/BMD (N = 6 or 7), and UA (N = 15 or 16) subjects, and analyzed alternative splicing by RT-PCR and gel electrophoresis. (**a**) Representative gel images showing alternative splicing of *INSR* exon 11, *MBNL2* exon 6, *SOS1* exon 25, *MBNL1* exon 7, *CLASP1* exon 20, *MAP3K4* exon 17, *NFIX* exon 7, *NCOR2* exon 45a, *VPS39* exon 3, and *MAPT* exons 2 and 3. PCR cycle number was 36 (*INSR*, *MBNL2*, *SOS1*, *CLASP1*, *MAP3K4*, *NFIX*, *NCOR2*, *VPS39*) or 37 (*MBNL1*, *MAPT*). Transcript name is shown on the left and target exon/intron on the right. Due to bands that were faint or absent, some samples required re-analysis of one or more transcripts using 38 or 40 cycles of PCR. Control UA muscle tissue cDNA, prepared from commercially available skeletal muscle tissue total RNA, was diluted 1:100 and amplified in the same PCR

reaction as urine samples. “L” = DNA ladder. “bp” = base pairs. (**b**) Individual data points represent quantification of splicing of all individual urine samples examined. Error bars indicate mean ± s.e.m. ** P = 0.0013 (*MBNL2*, DM1 vs. DMD/BMD) and P = 0.0013 (*MAP3K4*, DM1 vs. DMD/BMD); * P = 0.018 (*CLASP1*, DM1 vs. DMD/BMD), P = 0.019 (*MAPT*, DM1 vs. UA), and mean difference 12.3, 95% CI of difference 1.1 to 23.5 (*MBNL2*, DM1 vs. UA); a difference in splicing between the DM1 and UA groups fell short of statistical significance for *MAP3K4* (mean difference = 11.3, 95% CI of difference -1.8 to 24.5); one-way ANOVA.

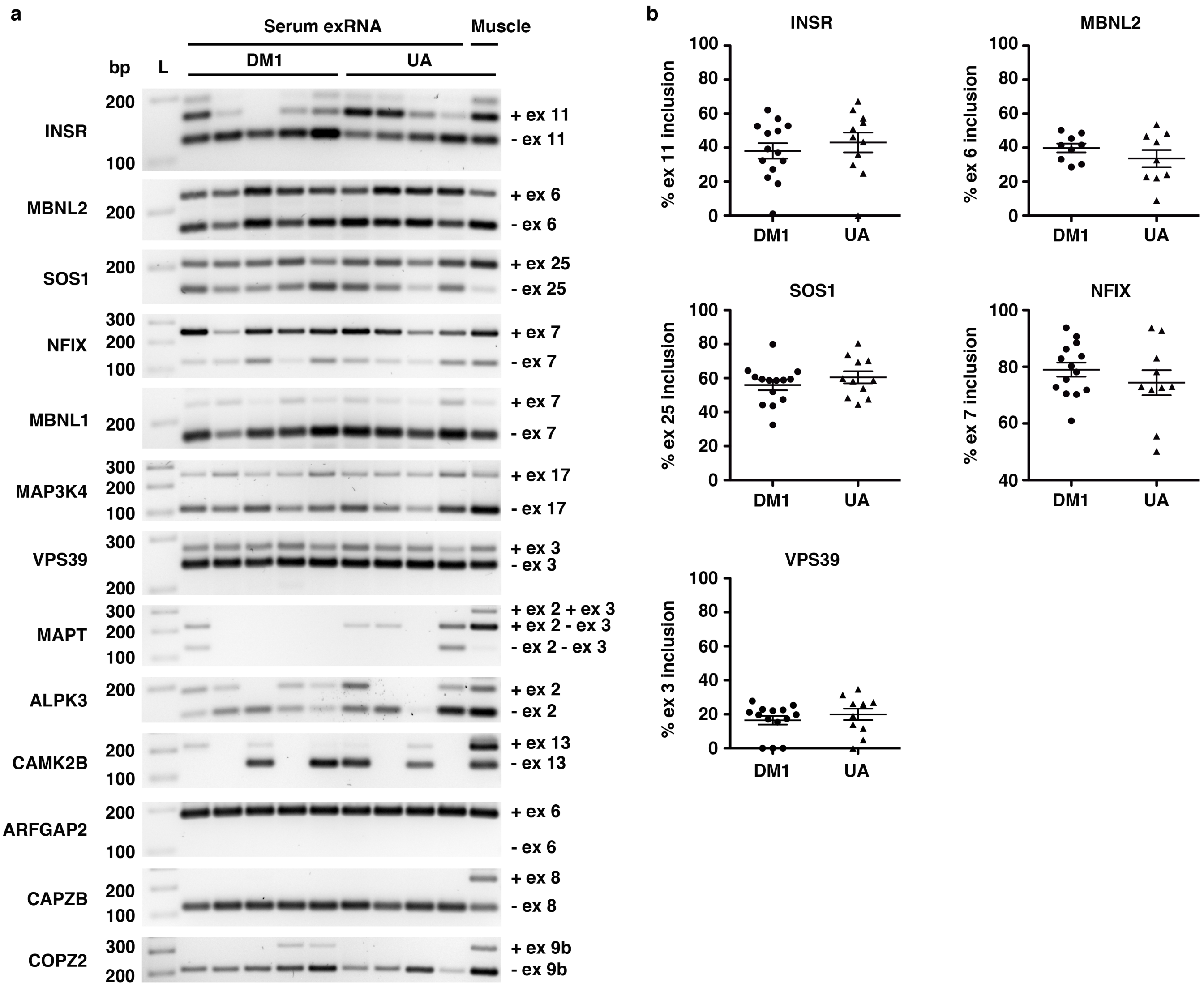

Supplementary Figure 6 Alternative splicing of exRNA in human serum. (**a**) We used RT-PCR to screen splicing in DM1 (N = 5) and UA control (N = 4) serum exRNA samples of several transcripts mis-spliced in DM1 muscle biopsies[2]. Transcript name is shown on the left and target exon/intron on the right. Examination of *VPS39* in an additional 9 DM1 and 7 UA samples and *ALPK3* in an additional 4 DM1 and 3 UA samples also showed no difference in splicing patterns by genotype. Alternative splicing patterns in serum appeared similar in DM1 and UA for all transcripts tested. cDNA made from commercially available skeletal muscle tissue total RNA served as a control. "L" = DNA ladder. "bp" = base pairs. (**b**) Quantification of alternative splicing by RT-PCR and gel electrophoresis of *INSR* exon 11 (N = 14 DM1, 10 UA), *MBNL2* exon 6 (N = 9 DM1, 9 UA), *SOS1* exon 25 (N = 14 DM1, 12 UA), *NFIX* exon 7 (N = 14 DM1, 12 UA), and *VPS39* exon 3 (N = 14 DM1, 11 UA) in human serum exRNA. Individual data points represent quantification of splicing of all serum samples examined. Error bars indicate mean ± s.e.m.

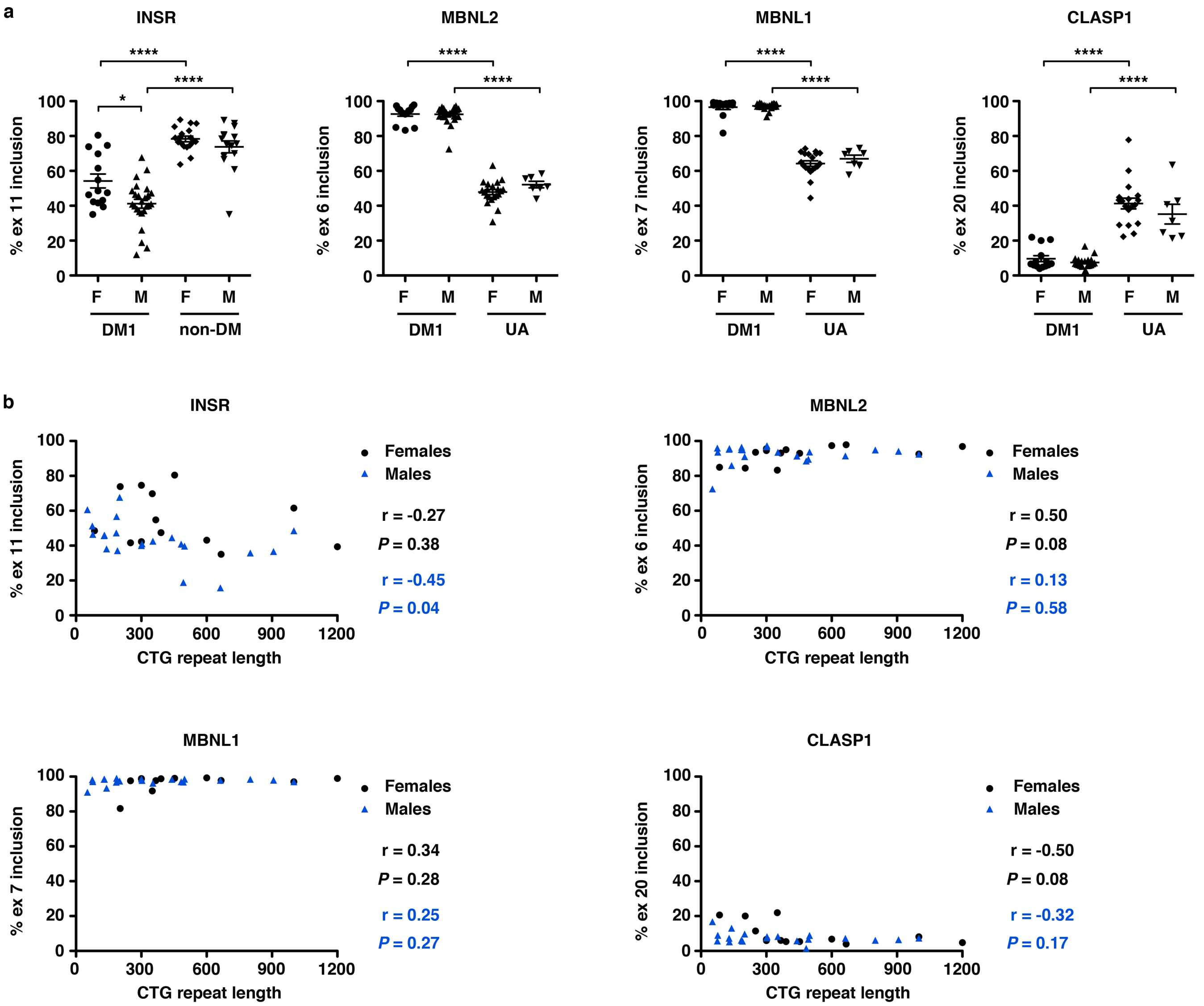

Supplementary Figure 7 Alternative spicing patterns in urine exRNA samples vs. biologic sex and CTG repeat length. (**a**) Percent exon inclusion by ddPCR of the transcripts *INSR*, *MBNL2*, *MBNL1*, and *CLASP1* in urine exRNA from females (F; N = 14 DM1, 18 UA) and males (M; N = 25 DM1, 7 UA) is shown. For *INSR*, splicing data from DMD/BMD subjects (N = 8, all male) also are combined with the UA group to form a single "non-DM" group. *MAP3K4* results are shown in Figure 7a. Error bars indicate mean ± s.e.m. **** $P < 0.0001$ female DM1 vs. female UA, and male DM1 vs. male UA or non-DM group; one-way ANOVA. (**b**) Of the 40 DM1 subjects examined, 34 (N = 13 females, 21 males) had genetic testing information that specified the number of CTG repeats available in the medical record. Shown is exon inclusion of *INSR*, *MBNL2*, *MBNL1*, and *CLASP1* in urine exRNA vs. the number of CTG repeats for each individual. The correlation coefficient r and *P* value for each transcript are indicated for females (black circles) and males (blue triangles). *MAP3K4* results are shown in Fig. 7b.

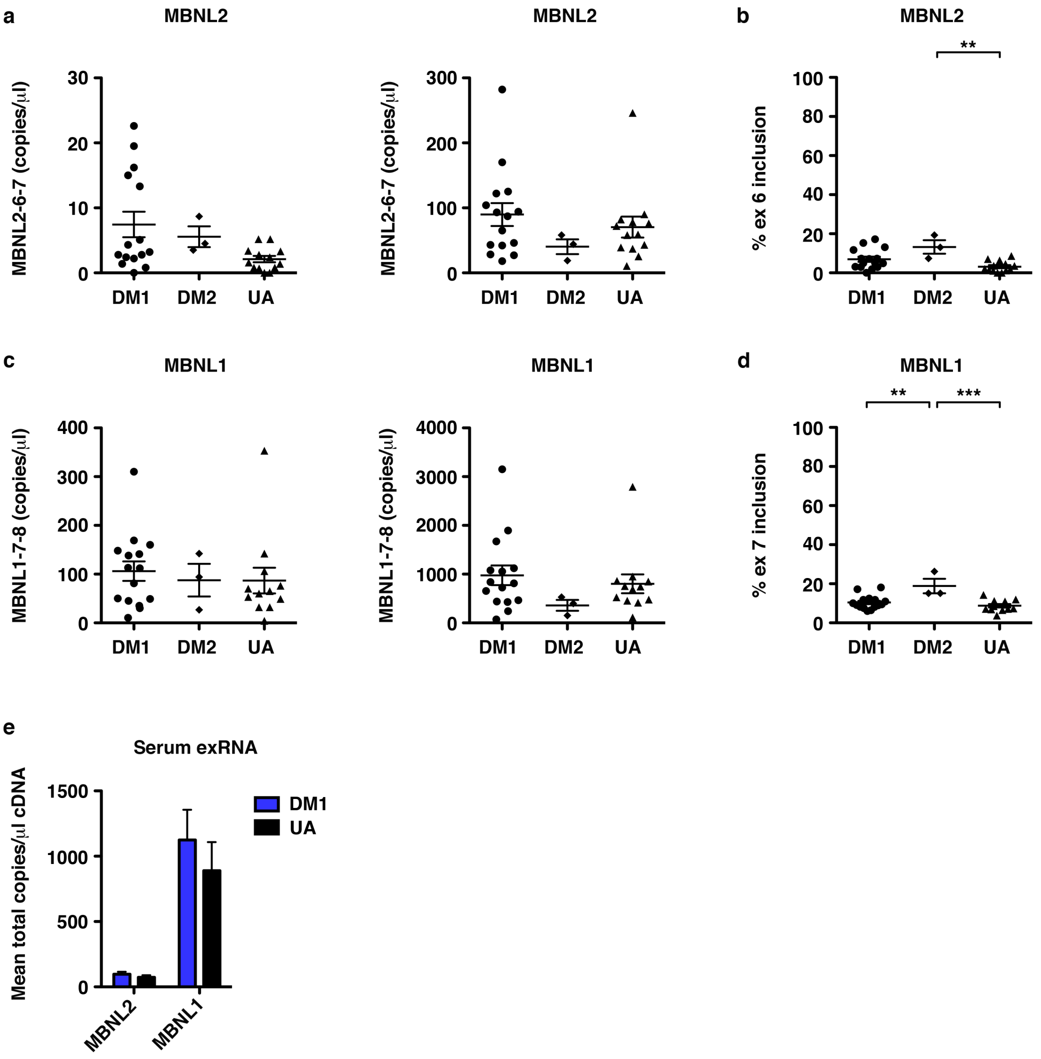

Supplementary Figure 8 Droplet digital PCR (ddPCR) analysis of alternative splicing in human serum exRNA. We used ddPCR to examine alternative splicing of *MBNL2* exon 6 and *MBNL1* exon 7 in serum exRNA of DM1 (N = 14 or 15), DM2 (N = 3), and UA (N = 12 or 13) individuals. Individual data points represent the mean of duplicate assays for each sample. (**a**) Quantification of *MBNL2* splice products that include exon 6 (MBNL2-6-7; left) and exclude exon 6 (MBNL2-5-7; right) in serum exRNA as transcript copies per micoliter of cDNA. Error bars = mean ± s.e.m. (**b**) Calculation of *MBNL2* exon 6 inclusion percentage using data in (**a**). Error bars = mean ± s.e.m. ** $P = 0.003$ (% exon 6 inclusion, DM2 vs. UA); one-way ANOVA. (**c**) Quantification of *MBNL1* splice products that include exon 7 (MBNL1-7-8; left) and exclude exon 7 (MBNL1-6-8; right) in serum exRNA as transcript copies per micoliter of cDNA. (**d**) Calculation of *MBNL1* exon 7 inclusion percentage using data in (**c**). Error bars = mean ± s.e.m. *** $P = 0.0008$ (% exon 7 inclusion, DM2 vs. UA); ** mean difference 8.4, 95% CI of difference 2.6 to 14.2 (% exon 7 inclusion, DM1 vs. DM2); one-way ANOVA. (**e**) Mean total copies/µl cDNA (exon inclusion + exclusion values from [**a**] and [**c**]) for *MBNL2* and *MBNL1* in serum exRNA. Error bars indicate mean ± s.e.m.

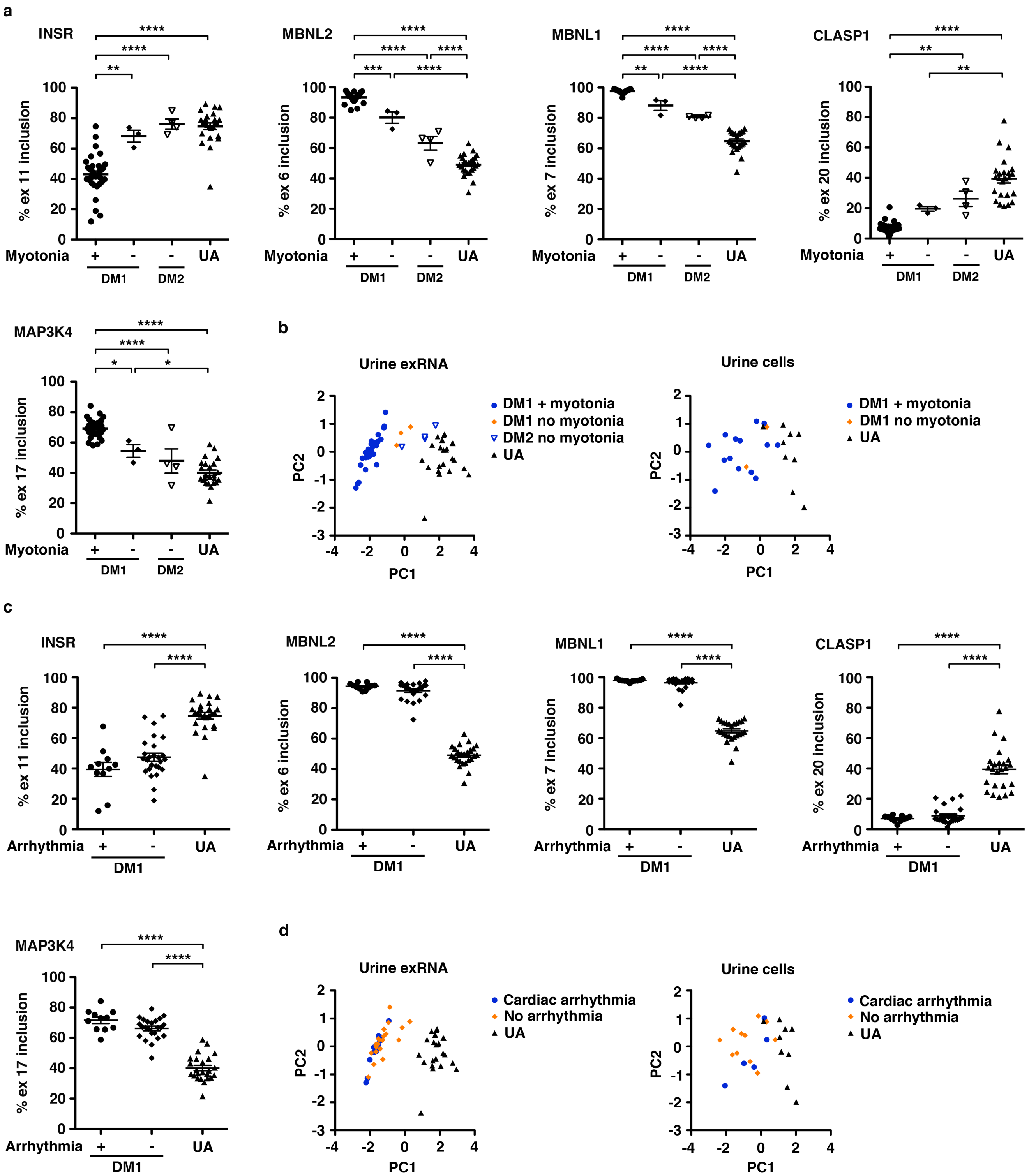

Supplementary Figure 9 Urine exRNA alternative splicing patterns as a function symptoms in DM. (**a**) ddPCR quantification of alternative splicing of *INSR*, *MBNL2*, *MBNL1*, *CLASP1*, and *MAP3K4* as % exon inclusion in urine exRNA of DM1 or DM2 individuals based on the presence ("+"; N = 34 DM1) or absence ("-"; N = 3 DM1; N = 4 DM2) of grip myotonia on physical examination. Values from UA controls also are shown. **** $P < 0.0001$; *** mean difference 13.4, 95% CI of difference 4.9 to 21.9 (*MBNL2*, DM1 + myotonia vs. DM1 no myotonia); ** mean difference 25.1, 95% CI of difference 5.8 to 45.3 (*INSR*, DM1 + myotonia vs. DM1 no myotonia), mean

difference 9.6, 95% CI of difference 2.3 to 16.8 (*MBNL1*, DM1 + myotonia vs. DM1 no myotonia), mean difference 19.9, 95% CI of difference 4.4 to 35.4 (*CLASP1*, DM1 no myotonia vs. UA), and mean difference 18.9, 95% CI of difference 5.5 to 32.3 (*CLASP1*, DM1 + myotonia vs. DM2 no myotonia); * mean difference 14.9, 95% CI of difference 1.8 to 28.0 (*MAP3K4*, DM1 + myotonia vs. DM1 no myotonia), and mean difference 14.3, 95% CI of difference 1.0 to 27.6 (*MAP3K4*, DM1 no myotonia vs. UA); one-way ANOVA. (**b**) ddPCR composite biomarker scores in urine exRNA (left) and urine cells (right), displayed as a function of grip myotonia. (**c**) ddPCR quantification of alternative splicing of *INSR*, *MBNL2*, *MBNL1*, *CLASP1*, and *MAP3K4* as % exon inclusion in urine exRNA of DM1 individuals that have cardiac arrhythmia ("+"; N = 11), defined as documented atrial fibrillation/flutter in the medical record, use of a pacemaker, and/or use of an automated implantable cardiac defibrillator (AICD), and those who have no known cardiac arrhythmia ("-"; N = 26). Values from UA controls also are shown. **** $P < 0.0001$; one-way ANOVA. (**d**) ddPCR composite biomarker scores in urine exRNA (left) and urine cells (right), displayed as a function of cardiac arrhythmia. All error bars indicate mean ± s.e.m.

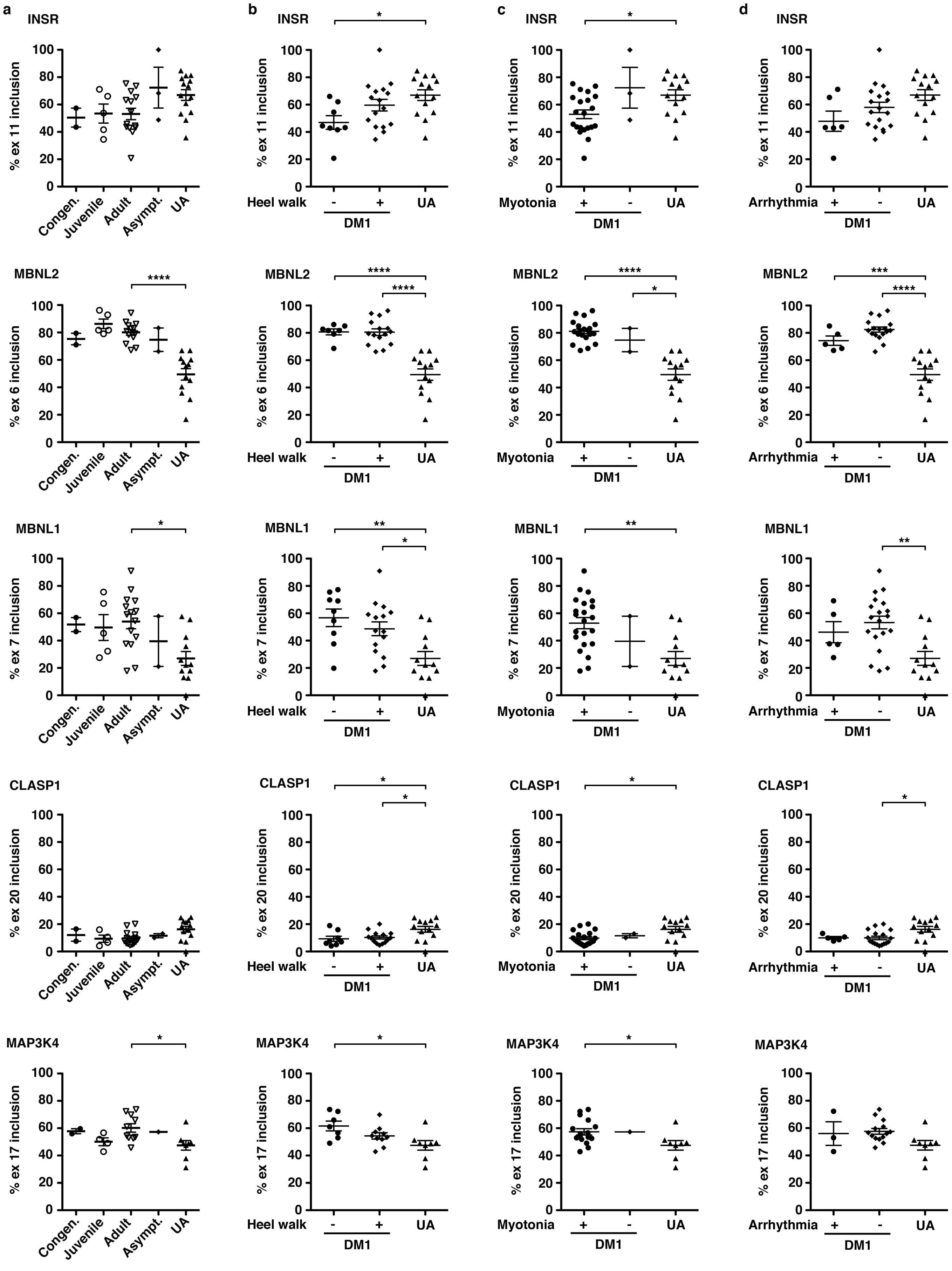
a
b
c
d
INSR
MBNL2
MBNL1
CLASP1
MAP3K4
% ex 11 inclusion
% ex 6 inclusion
% ex 7 inclusion
% ex 20 inclusion
% ex 17 inclusion
Congen.
Juvenile
Adult
Asympt.
UA
Heel walk
Myotonia
Arrhythmia
DM1
*
**
***
****

Supplementary Figure 10 Alternative splicing patterns in urine cell RNA as a function of symptoms in DM. We used ddPCR to examine alternative splicing of *INSR* exon 11, *MBNL2* exon 6, *MBNL1* exon 7, *CLASP1* exon 20, and *MAP3K4* exon 17 in urine cells of individuals with DM1 (N = 17 to 24) as a function of symptoms. Splicing in urine cells of UA individuals (N = 8 to 14) served as a control. (**a**) Splicing of all five transcripts as a function of age of symptom onset: congenital (N = 2), juvenile (N = 4 or 5), adult (N = 13), and asymptomatic DM1 (N = 2 or 3). **** $P < 0.0001$; * $P = 0.02$; one-way ANOVA. (**b**) Splicing of all five transcripts as a function of a DM1 individual's ability to walk at least 5 steps on the heels while maintaining ankle dorsiflexion (a functional measure of ankle strength) ("+"; N = 10 to 16), and DM1 patients who were unable ("-"; N = 7 to 9). **** $P < 0.0001$; ** $P = 0.002$; * $P = 0.02$; one-way ANOVA. (**c**) Splicing of all five transcripts in samples from DM1 individuals with grip myotonia ("+") (N = 20 to 22) or without grip myotonia ("-") (N = 2 or 3). **** $P < 0.0001$; ** $P = 0.002$ (*MBNL1*, DM1 + myotonia vs. UA); * $P = 0.02$; one-way ANOVA. (**d**) Splicing of all five transcripts in DM1 individuals with cardiac arrhythmia ("+"; N = 3 to 6), defined as documented atrial fibrillation/flutter in the medical record, use of a pacemaker, and/or use of an automated implantable cardiac defibrillator (AICD), and those who have no known cardiac arrhythmia ("-"; N = 12 to 17). **** $P < 0.0001$; *** mean difference 24.9, 95% CI of difference 10.2 to 39.5 (*MBNL2,* DM1 + arrhythmia vs. UA); ** $P = 0.003$; * $P = 0.02$; one-way ANOVA. All error bars indicate mean ± s.e.m.

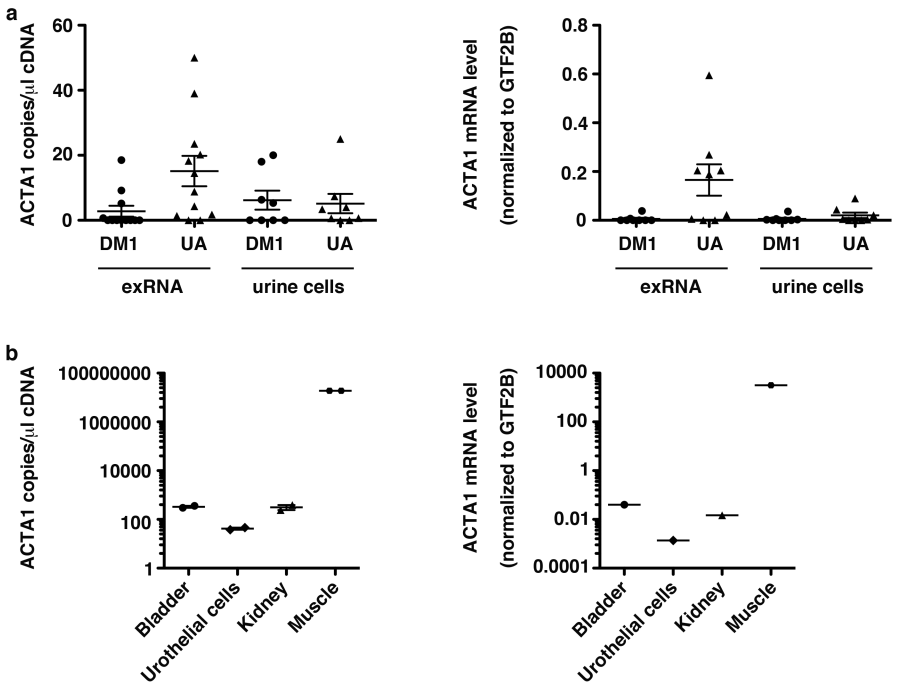

Supplementary Figure 11 Skeletal muscle actin (*ACTA1*) gene expression in urine exRNA, urine cells, urinary tract tissues, and skeletal muscle tissue. (**a**) ddPCR analysis of *ACTA1* expression in urine exRNA (N = 17 DM1, 14 UA) and urine cell total RNA (N = 14 DM1, 9 UA) in copies per microliter (*µ*l; left) of cDNA and normalized to reference gene *GTF2B* (right). Note that most of the individual data points overlie the x-axis of both graphs. *GTF2B* copies per *µ*l of cDNA are shown in Fig. 1. Individual data points represent the mean of duplicate assays for each sample. Error bars indicate ± s.e.m. (**b**) ddPCR analysis of *ACTA1* expression in commercially available total RNA from human bladder, urothelial cells (transitional epithelial cells that line the inside of the ureters and bladder), kidney, and skeletal muscle (see methods) in copies per *µ*l of cDNA for each replicate (left) and normalized to reference gene *GTF2B* (right). Individual data points represent the mean of duplicate assays for each sample. Error bars indicate mean ± s.e.m.

a

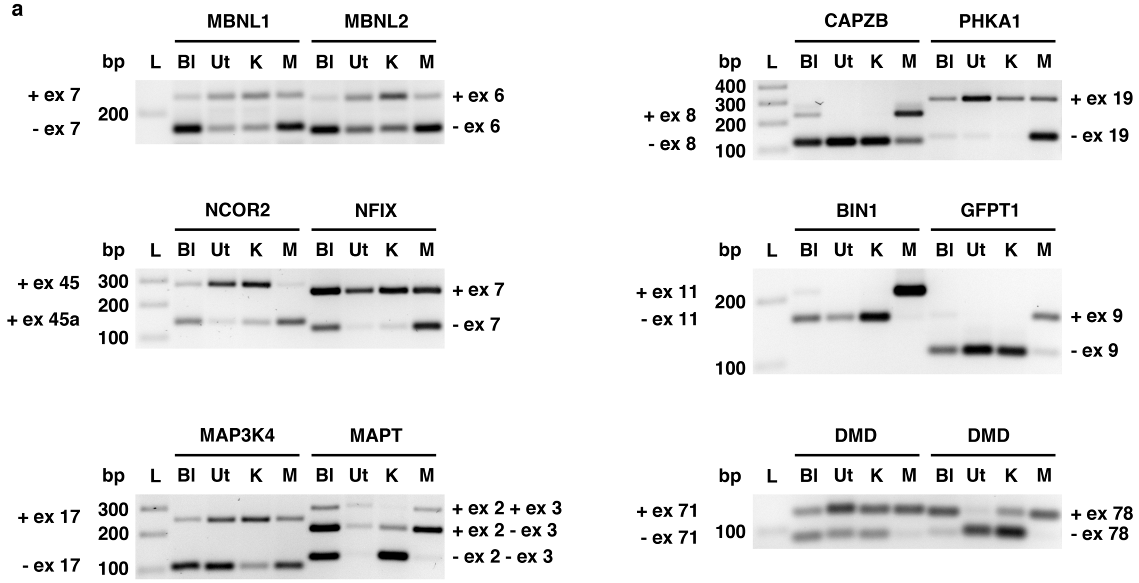

b

Transcript	Exon	Bladder	Urothelial	Kidney	Muscle	Urine ex-RNA (s.e.m.)
MBNL1	7	8.7	47.3	53.8	20.0	48.3 (2.9)
MBNL2	6	5.2	33.0	49.4	13.8	52.7 (1.3)
MAP3K4	17	15.3	32.7	65.5	28.3	41.3 (1.2)
MAPT	2, 3	57.5	78.7	15.0	95.2	53.5 (3.0)
NCOR2	45a	25.2	89.4	81.2	10.6	75.7 (2.1)
NFIX	7	67.8	92.2	91.2	48.1	80.4 (1.8)
CAPZB	8	14.5	< 1	< 1	60.2	< 1
PHKA1	19	77.5	94.1	95.7	27.4	95.5 (4.2)
BIN1	11	8.1	2.1	3.3	98.5	1.0 (0.4)
GFPT1	9	6.0	< 1	1.2	79.9	< 1
DMD	71	41.8	70.9	68.3	93.1	77.3 (3.8)
DMD	78	86.8	5.4	15.1	97.3	< 1

Supplementary Figure 12 Alternative splicing in human bladder, urothelial cells, kidney, and skeletal muscle. (**a**) Urine EVs are derived from the kidney and urinary tract[3], and splicing of transcripts derived from these tissues may explain the different splicing pattern of several transcripts in urine exRNA than in skeletal muscle (Fig. 2, Supplementary Fig. 3; Supplementary Table 5). To determine if the urinary tract may be the predominant source of urine exRNA, we screened twelve alternative splice events in commercially available total RNA from normal human bladder (B), urothelial (Ut; transitional epithelial) cells, kidney (K) and skeletal muscle (M) tissue by RT-PCR using random primers and 26 or 28 cycles for each transcript. "L" = DNA ladder. "bp" = base pairs. (**b**) Percent exon inclusion of each transcript shown in (**a**) and mean percent exon inclusion in UA exRNA ± s.e.m.

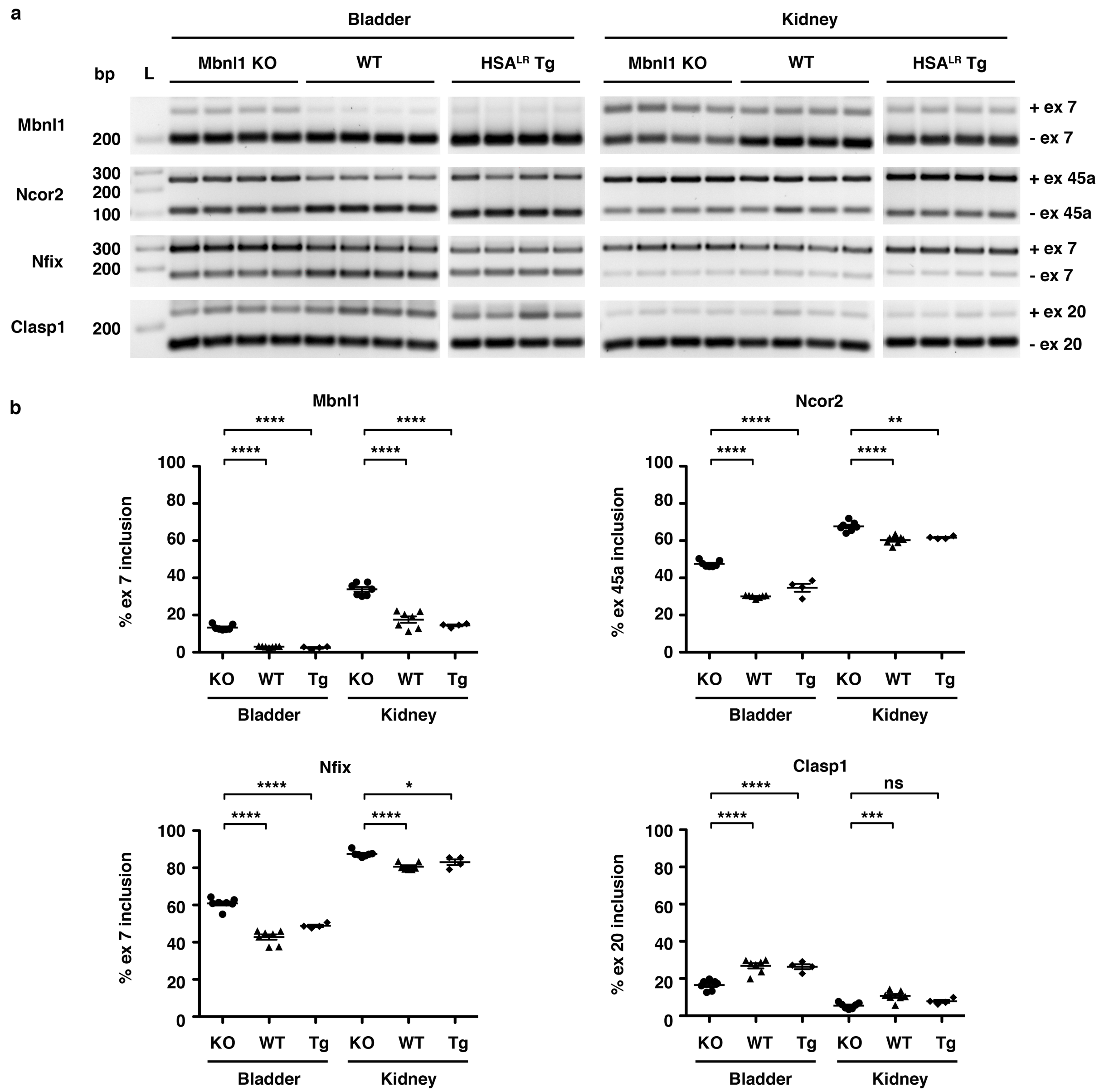

Supplementary Figure 13 Alternative splicing in mouse bladder and kidney tissue. We examined alternative splicing of *Mbnl1*, *Ncor2*, *Nfix*, and *Clasp1* transcripts in bladder and kidney tissues in the *Mbnl1* knockout[4] (*Mbnl1*$^{\Delta E3/\Delta E3}$) mouse model[4] of DM1 ('KO"; N = 7), the Human Skeletal Actin - Long Repeat (HSA^{LR}) transgenic mouse[5] model of DM1 ("Tg"; N = 4), and wild-type controls ("WT"; N = 7) by RT-PCR and gel electrophoresis. By design, DM1-associated mis-regulation of alternative splicing in the HSA^{LR} model is restricted to skeletal muscle[5]. (**a**) Representative splicing patterns of each transcript. "L" = DNA ladder. "bp" = base pairs. (**b**) Quantification of exon inclusion for each transcript in each mouse tissue examined. Error bars indicate mean ± s.e.m. **** $P <$ 0.0001 (all four transcripts, KO vs. WT and KO vs. Tg; bladder), (*Mbnl1*, *Ncor2,* and *Nfix*, KO vs. WT; kidney) (*Mbnl1*, KO vs. Tg; kidney); *** $P = 0.0013$ (*Clasp1*, KO vs. WT; kidney); ** P = mean difference 6.0, 95% CI of difference 2.3 to 9.7 (*Ncor2*, KO vs Tg; kidney); * P = mean difference 4.4, 95% CI of difference 0.9 to 7.9 (*Nfix*, KO vs. Tg; kidney); ns = non-significant; one-way ANOVA.

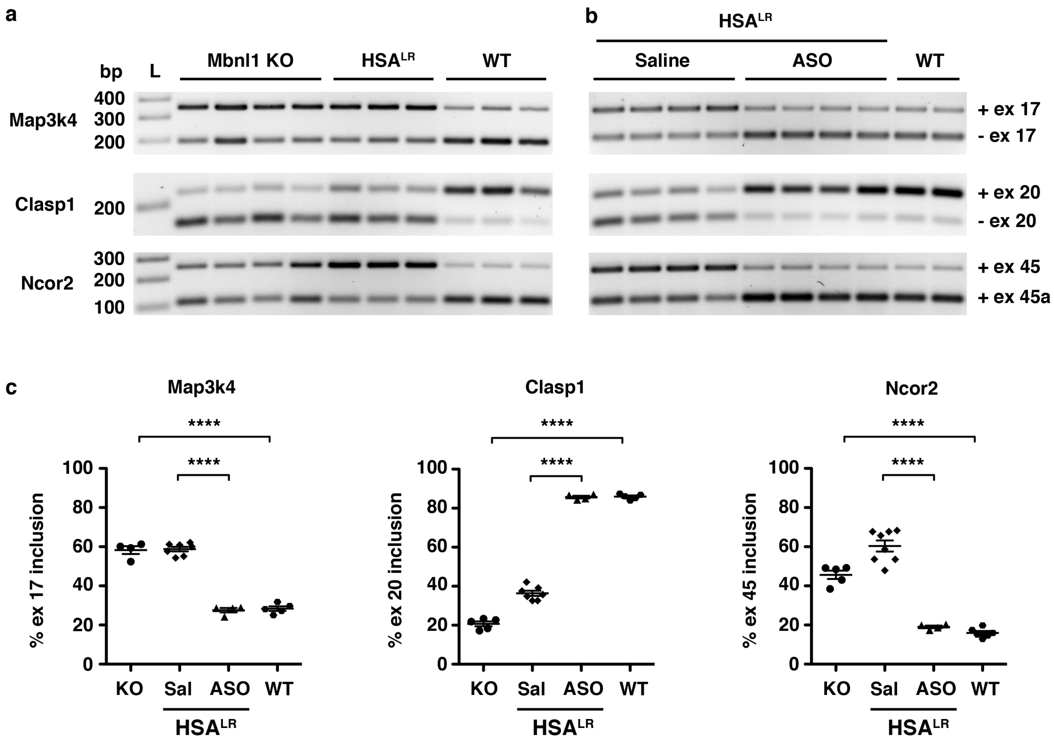

Supplementary Figure 14 Regulation of *Map3k4*, *Clasp1*, and *Ncor2* splicing in skeletal muscle by MBNL1 protein and response to ASO treatment. (**a**) We used RT-PCR to analyze alternative splicing of *Map3k4*, *Clasp1*, and *Ncor2* in gastrocnemius muscles from 2 mouse models of DM1, the *Mbnl1* knockout[4] (*Mbnl1*$^{\Delta E3/\Delta E3}$; N = 4) and HSA^{LR} transgenic[5] (N = 3), and FVB wild-type (N = 3). "L" = DNA ladder. "bp" = base pairs. (**b**) We treated HSA^{LR} with either saline or ASO 445236 [6] (N = 4 each) using a dose of 25 mg/kg twice weekly for 4 weeks, and analyzed alternative splicing in quadriceps muscles from these mice by RT-PCR. Gastrocnemius muscles from untreated FVB wild type (N = 2) served as controls. (**c**) Quantification of alternative exon 17 (*Map3k4*), exon 20 (*Clasp1*), and exon 45a (*Ncor2*) splicing in each individual replicate from (**a**) and (**b**). Error bars indicate mean ± s.e.m. **** $P < 0.0001$ (all three transcripts, saline vs. ASO) and (all three transcripts, KO vs. WT); one-way ANOVA. These data demonstrate that alternative splicing of *Map3k4*, *Clasp1*, and *Ncor2* is regulated by MBNL1 protein, and that mis-splicing of these transcripts in the HSA^{LR} mouse model of DM1 is rescued by ASO treatment, similar to ASO rescue of other alternatively spliced exons that also are regulated by MBNL1 protein[2, 6].

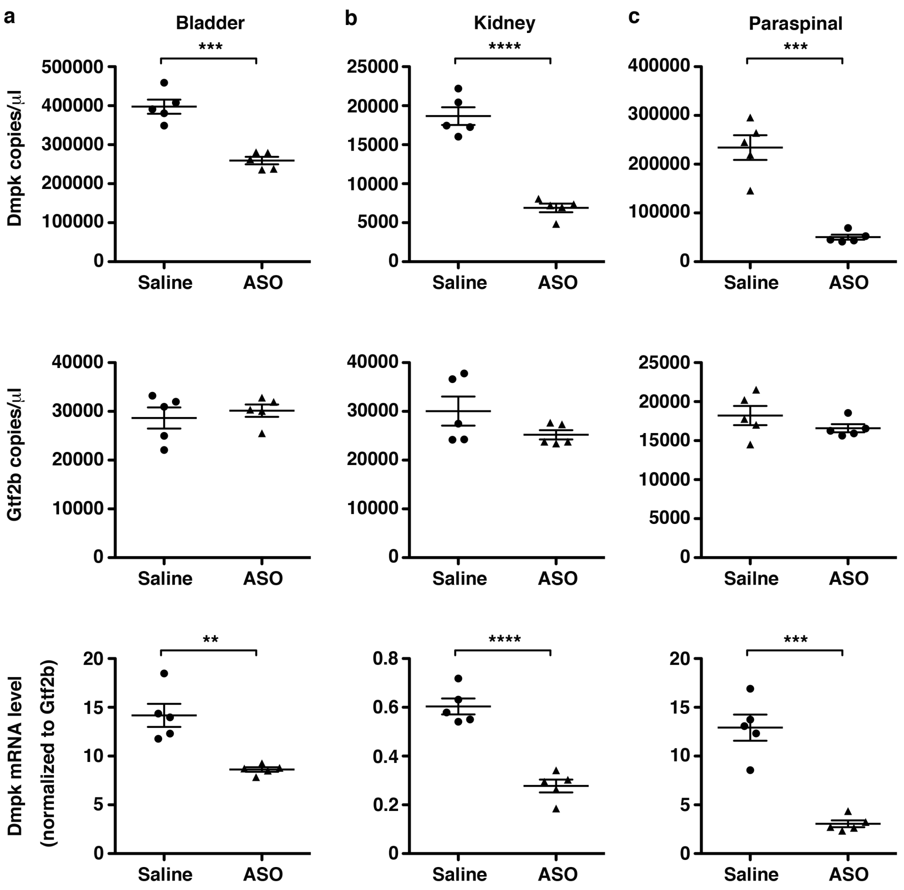

Supplementary Figure 15 Therapeutic ASO drug activity in urinary tract and muscle tissues. We treated FVB wild-type mice with saline or a therapeutic ASO (N = 5 each) that induces RNase H cleavage of *Dmpk* mRNA[7]. ASO dose was 25 mg/kg twice weekly for four weeks (eight total doses). Bladder, kidney, and paraspinal muscle tissues were collected one week after the final dose. The ASO target sequence in *Dmpk* transcripts is identical in mouse, monkey, and human. Shown is expression of *Dmpk* (upper) and reference gene *Gtf2b* (middle) by ddPCR in copies per microliter (*µ*l) of cDNA, and *Dmpk* expression normalized to *Gtf2b* (lower) in bladder (**a**), kidney (**b**), and paraspinal muscle (**c**) tissues. Individual data points represent the mean of duplicate assays for each sample. Error bars indicate mean ± s.e.m. **** $P < 0.0001$ (*Dmpk* copies/ *µ*l, saline vs. ASO; kidney) and (*Dmpk* normalized to *Gtf2b*, saline vs. ASO; kidney); *** $P = 0.001$ (*Dmpk* copies/ *µ*l, saline vs. ASO; bladder and paraspinal muscle) and (*Dmpk* normalized to *Gtf2b*, saline vs. ASO, paraspinal muscle); ** $P = 0.0017$ (*Dmpk* normalized to *Gtf2b*, saline vs. ASO, bladder); two-tailed t-tests.

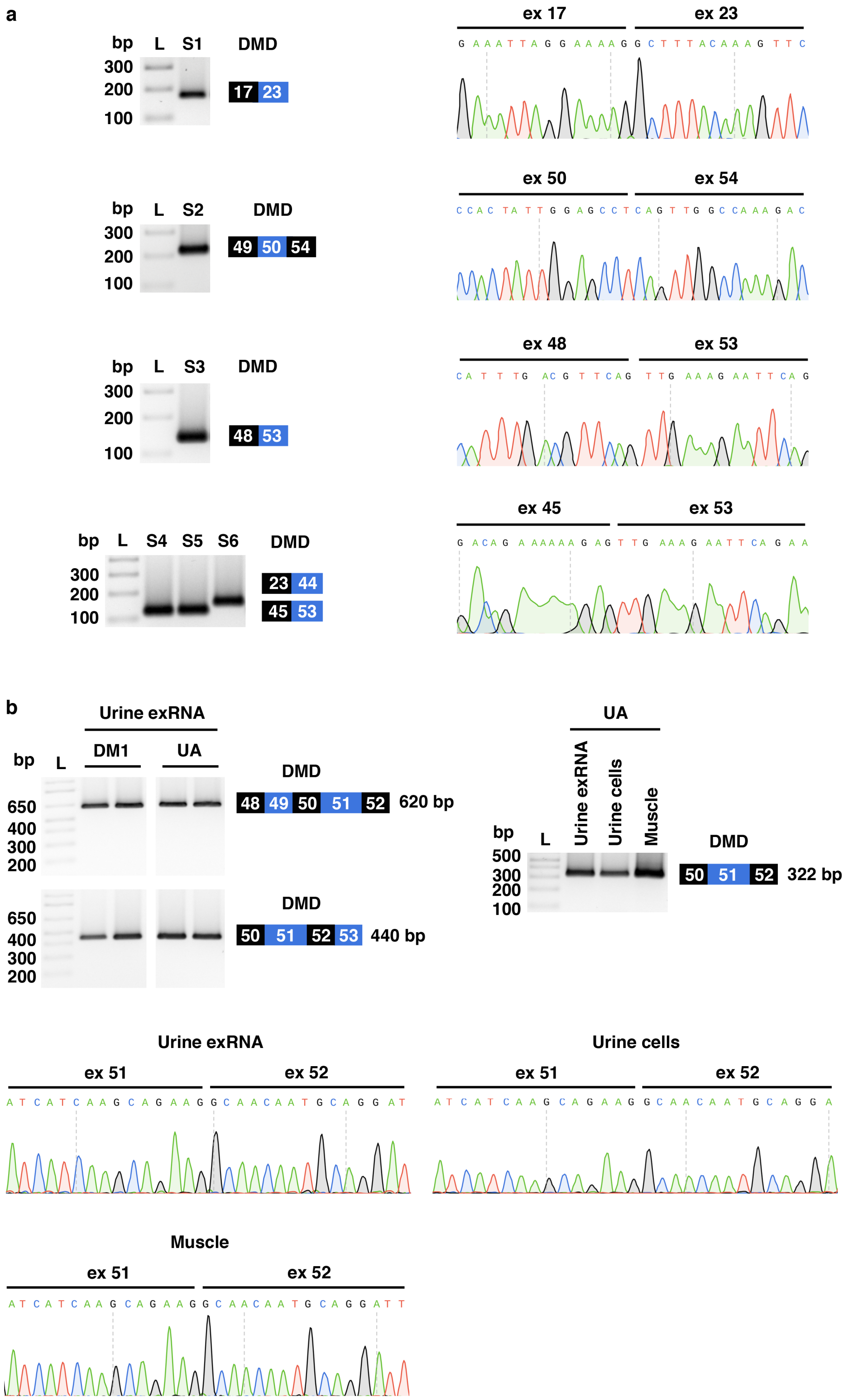

Supplementary Figure 16 Patient-specific *DMD* deletion transcripts and expression of *DMD* exon 51 in urine exRNA and urine cells. (**a**) Using mutation-specific primers and RT-PCR (left), we examined urine exRNA from six DMD subjects with frame-shifting deletions of exons 18 - 22 (Subject 1; S1), exons 51 - 53 (S2), exons 49 - 52 (S3), exons 46 - 52 (S4 and S5), or exons 24 - 43 (S6). Deletions in S2, S3, S4, and S5 are candidates for exon skipping ASOs to restore the *DMD* reading frame. Sequencing the PCR products (right) confirmed the presence of each deletion transcript. The boxes show the exons amplified. “bp” = base pairs; “L” = DNA ladder. (**b**) To

identify *DMD* exon 51-containing transcripts in human urine, we examined urine exRNA from DM1 and UA individuals, and urine cells from UA individuals (right) using RT-PCR and primers targeting exons 48 and 52, exons 50 and 53, and exons 50 and 52 DM1, with expected amplicon sizes of 620 bp, 440 bp, or 322 bp. Total RNA from normal human skeletal muscle tissue served as a control. Gel electrophoresis of PCR products (left) showed no evidence of an alternative splice event, consistent with previous reports that *DMD* exon 51 is constitutively spliced[8, 9]. Sequencing of PCR products from urine exRNA, urine cells, and muscle (lower).

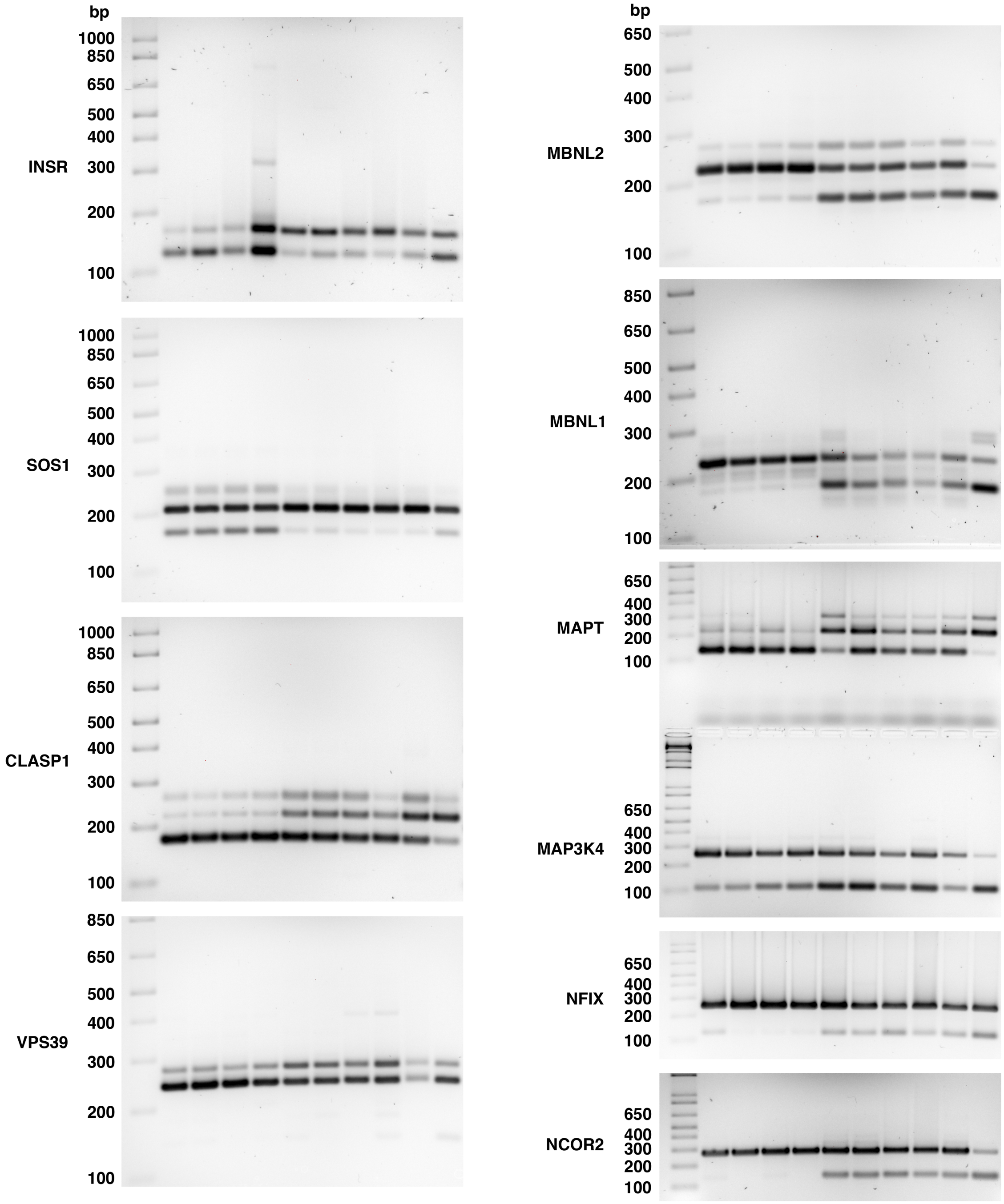

Supplementary Figure 17 Uncropped gel images of urine exRNA splicing outcomes of the ten transcripts shown in Figure 2. The molecular weight marker is the 1 Kb Plus DNA Ladder (Invitrogen). “bp” = base pairs.

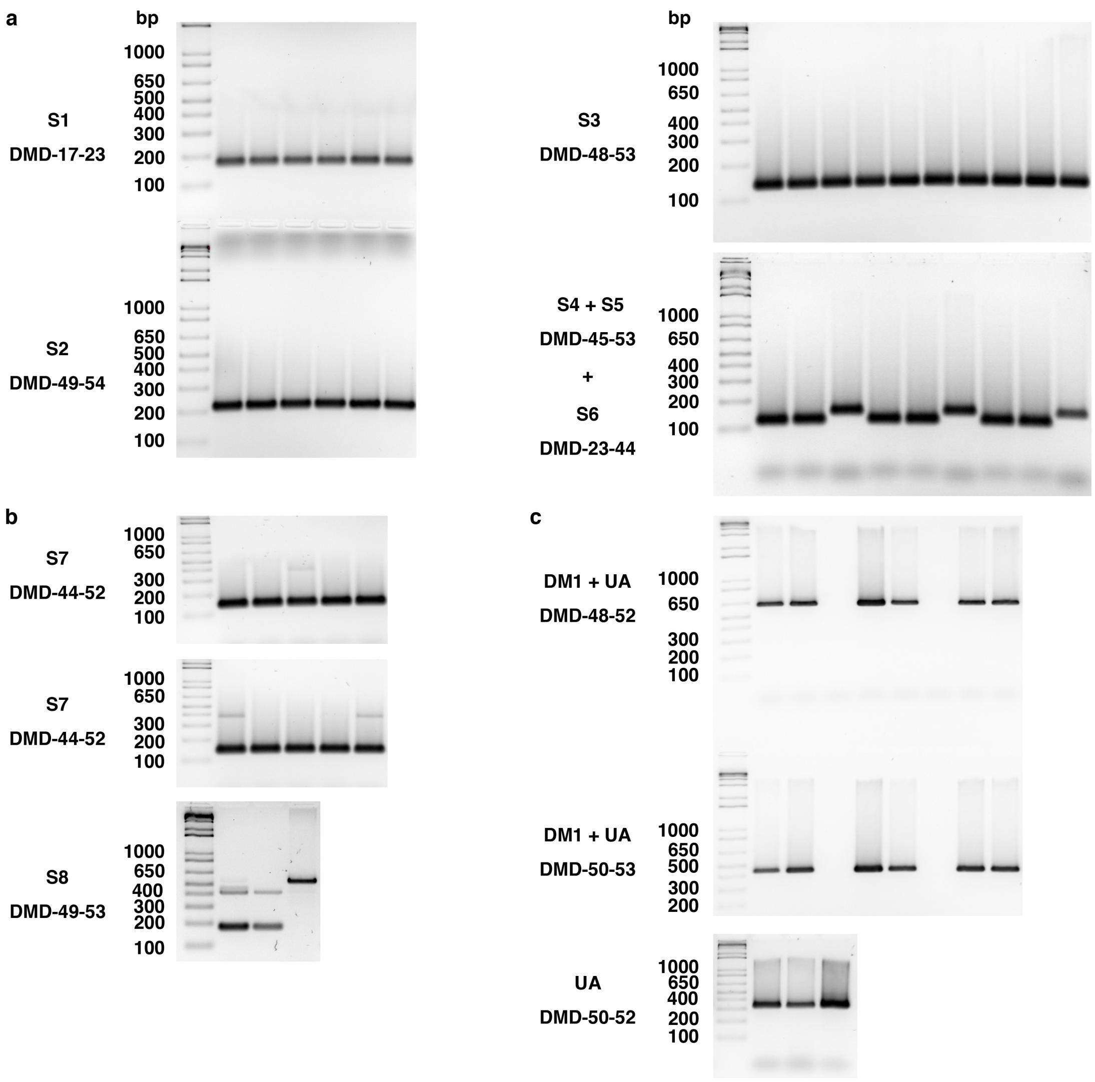

Supplementary Figure 18 Uncropped gel images of DMD deletion transcripts. (**a**) Subjects S1 through S6 shown in Supplementary Figure 16a. (**b**) Subjects S7 and S8 shown in Figure 10. (**c**) DM1 and UA subjects shown in Supplementary Figure 16b. The molecular weight marker is the 1 Kb Plus DNA Ladder (Invitrogen). “bp” = base pairs.

Gene Name	Systematic name	Sequence	Probe Name	Avg	Ctrl1	Ctrl2	Ctrl3	Ctrl4	Ctrl5	Ctrl7	Ctrl8
DMPK	NM_004409	TTTTGGATGCACTGAGACCCCGACATTCCTC GGTATTTATTGTCTGTCCCCACCTAGGAC	A_23_P50535	5.66	5.73	5.65	5.50	5.73	5.65	5.69	5.73
INSR	NM_000208	GTTCAGAGATCGTTCCTATACATTTCTGTTC ATCTTAAGGTGGACTCGTTTGGTTACCAA	A_23_P4764	4.87	4.87	5.00	4.56	4.85	4.98	4.92	4.64
MBNL2	NM_144778	ATCTTTCTGTAACACTTAAAGAATTCCCTCA TTCATTACCTTACAGTGTAAACAGGAGTC	A_24_P56317	4.60	4.50	4.37	5.44	4.53	4.48	4.30	4.53
SOS1	NM_005633	TTATTACCACCACGAGAACCTGTGAGGACAC CTGATGTTTTCTCAAGCTCACCACTACAT	A_23_P343808	4.77	4.50	4.81	4.92	4.80	4.98	4.59	4.56
CLASP1	NM_015282	TTATCAAGCGTAATGTTACACTTTAAAGGAC AGCAAATAAGAACTTTGTAGAATCCCACC	A_23_P311232	4.57	4.51	4.53	4.59	4.81	4.59	4.37	4.73
MBNL1	NM_021038	ATCCTTTCAAACCCTCATGACTGACAAAAAC TCCATGGGGCCAAATCTGCCTGAAGATCA	A_23_P357811	6.26	6.44	6.14	6.45	5.99	6.70	5.87	5.86
MAP3K4	NM_005922	AAAGATTAAGCCCTGAAGGAAAGGACTTCCT TTCTCACTGCCTTGAGAGTGACCCAAAGA	A_23_P42096	5.07	5.29	4.70	5.18	5.21	5.04	4.99	5.37
NFIX	NM_002501	ACCTGGTCATGGTGATTTTGTTTAAGGGGAT CCCCCTGGAAAGTACTGATGGGGAGCGGC	A_23_P165295	4.85	4.90	4.83	4.79	4.99	4.55	5.05	5.11
NCOR2	NM_006312	TTCGATGCGTATTCTGTGGCCGCCATTTGCG CAGGGTGGTGGTATTCTGTCATTTACACA	A_23_P203891	5.84	5.71	5.65	6.16	5.97	5.86	5.99	6.02
VPS39	NM_015289	GGCAAGAACAGCAGGACGCTGGTTTAAAAAT AACTCACCGCCAAACCTGTGGAGCAGTGT	A_24_P167825	5.08	5.55	4.66	4.73	4.80	5.38	5.34	4.99
MAPT	NM_016835	ACCAGTTCTCTTTGTAAGGACTTGTGCCTCT TGGGAGACGTCCACCCGTTTCCAAGCCTG	A_23_P207699	5.19	5.28	4.95	5.22	5.36	5.15	5.20	5.42
KIF13A	NM_022113	TCATATTCATTCCCTGGGATGTTTAGTTACC AGTTTTCCCAAAGTGTTCTGGTAGCATCT	A_23_P214111	4.69	4.51	4.72	4.66	4.75	4.67	4.83	4.63
DMD	NM_004019	AGAAAATATAGTCACAGGAAACTACTCACGT AAGTAGTAATGATTCTCAAGATCAAAGGG	A_23_P321860	4.44	4.45	4.44	4.45	4.30	4.73	4.27	4.39
CLCN1	NM_000083	TCCATCTTCCAGTCCCTGCTTCACTGCTTGC TGGGCAGAGCTCGCCCCACAAAGAAGAAA	A_23_P59772	6.90	6.85	6.93	6.78	7.05	6.88	6.90	7.26
ATP2A1	NM_173201	GTCCTCAAGATCTCACTGCCAGTCATTGGGC TCGACGAAATCCTCAAGTTCGTTGCTCGG	A_23_P72462	5.17	5.06	5.08	5.42	5.05	4.95	5.47	5.12
CAPZB	NM_004930	GCTGAACGAGATCTACTTTGGAAAAACAAAG GATATCGTCAATGGGCTGAGGTCTGTGCA	A_23_P126752	5.22	5.34	5.07	5.49	4.91	5.28	5.24	5.00
CACNA1S	NM_000069	TGGAGTCCTCCATGCCTGAGGACAGAAAGAG CTCCACACCAGGGTCTCTTCATGAGGAGA	A_23_P85765	5.80	5.72	5.79	5.76	5.85	5.83	5.84	5.68
CAMK2B	NM_172082	GATCATTAAGACCACGGAGCAGCTCATCGAG GCCGTCAACAACGGTGACTTTGAGGCCTA	A_23_P42882	4.79	4.87	4.54	4.60	4.67	5.17	4.88	5.39
COPZ2	NM_016429	GGTTCTTCAGTCTGCCAAGGAACAAATTAAA TGGTCGTTATTGAAATGAAGGCTGTGGAT	A_23_P101093	4.76	4.89	4.77	4.74	4.74	4.42	4.97	4.54
GFPT1	NM_002056	TGAAGGCATCCTTGCTGGTGAATTGAAACAT GGCCCTCTGGCTTTGGTGGATAAATTGAT	A_23_P44083	6.03	6.14	6.02	5.96	6.30	5.79	5.96	6.30
IMPDH2	NM_000884	TTGGACTCTTCCCAGGGAAATTCCATCTTCC AGATCAATATGATCAAGTACATCAAAGAC	A_24_P166042	5.00	4.76	5.28	4.57	5.00	5.29	5.09	4.94
ALPK3	NM_020778	TGTGTACCCCTTAGCAGGGTGTCTGGGGACT TACGCCTTTGGAATTGCTCTTCATTCAGA	A_23_P348728	5.95	5.78	5.92	6.03	6.03	5.71	6.22	5.83
ANK2	NM_001148	CCCCATCCTCTTTAACTATAAAGCTAATTTG TGACCAAAGATGGCATCCTTCATACTGGA	A_23_P133068	5.50	5.84	5.56	5.55	5.35	5.48	5.19	5.52
BIN1	NM_139346	AGCAAAGGGAAATCAAGAGGAGACCCCCAGG CAGAGGGGCGTTCTCCCAAAGATTAGGTC	A_23_P165333	5.63	5.78	5.64	5.49	5.53	5.69	5.65	5.38
NRAP	NM_198060	GGCCAGAGAGGAAGTTTGTTCACCAGAGACA GGCTTCAGATGGCTTTGATTTCGGCAAGC	A_23_P402765	4.58	4.60	4.49	4.19	4.74	4.84	4.64	4.77
OPA1	NM_130837	GTGCTTCCCAGCCTCACAATGTGGGAATTTG ACATAGGATGAGAGTCAGAGTATAGGTTT	A_23_P211797	6.54	6.54	6.60	6.62	6.43	6.57	6.50	6.55
PHKA1	NM_002637	GCTATGTTCAGAAAGATGCTTGGGTCCGAGA TAATGTGTACAGCATCTTGGCTGTGTGGG	A_32_P186121	5.26	5.80	5.30	5.39	5.20	4.89	4.97	5.78
UBE2D3	NM_181886	ATATAGCACTGAATAAATGATGCAAGTTGTC AATGGATGAGTGATCAACTAATAGCTCTG	A_24_P363005	6.05	6.42	6.19	6.43	5.54	6.11	5.60	5.81
PDLIM3	NM_014476	AAACATACACTTAGCTATGTTTTGCAACTCT TTTTGGGGCTAGCAATAATGATATTTAAA	A_23_P110403	4.51	4.49	4.35	4.51	4.46	4.58	4.67	4.63
LDB3	NM_007078	TTTTTTGCCTGTGTGAATTCTACTTTTTAGC AAAAATAAAGCCCCCCAAAGGATGTGCAA	A_32_P98227	5.11	5.26	5.14	5.19	5.06	5.18	4.81	5.48
TTN	NM_133378	CTGACAACCCTGATCATCATGGACGTACAGA AACAAGATGGTGGACTTTATACCCTGAGT	A_23_P85269	4.66	4.78	4.65	4.59	4.57	4.68	4.69	4.68
FHOD1	NM_013241	ATCATGGACCTTCTGGTGCAGTCAGTGACCA AGAGCAGTCCTCGTGCCTTAGCTGCTAGG	A_23_P37778	5.46	5.48	5.23	5.52	5.46	5.39	5.66	5.54
TBC1D15	NM_022771	CAACACAGATACCAGTGTCCTCAGATGTCTG CAGATTAACACCTGCATGATCACTGTTCT	A_23_P139558	5.10	5.10	4.86	5.00	5.12	5.30	5.22	5.09
RYR1	NM_000540	GAGTCTTATGTCTGGAAGATGTACCAAGAGA GATGTTGGGATTTCTTCCCAGCTGGTGAT	A_23_P78867	4.76	4.89	4.81	4.73	4.72	4.80	4.62	4.69
DTNA	NM_001392	TCGGACGGTGCTTTTGGTGGATGCGTCTAGA TGGATAACATGACTTCTTCTACCCTAAAA	A_23_P208158	4.82	4.62	4.87	4.91	4.91	4.66	4.94	4.89
TNNT2	NM_000364	AACAGGAGGAAGGCTGAGGATGAGGCCCGGA AGAAGAAGGCTTTGTCCAACATGATGCAT	A_23_P34700	5.28	5.41	5.35	5.17	5.14	5.07	5.55	5.15
FXR1	NM_001013439	AGTCTTTACATCGCACTTTCAGTTCCTCCAT TTGGAATTCATAAAGGGGAGGGATCCTGA	A_23_P132784	5.47	5.51	5.45	5.85	5.16	5.54	5.33	5.20
CKM	NM_001824	ACACTCGGAGCTTGTGCTTTGTCTCCACGCA AAGCGATAAATAAAAGCATTGGTGGCCTT	A_23_P50250	4.75	4.88	4.55	4.46	4.81	4.81	4.99	4.88
ACTA1	NM_001100	CCGCAGTCACTTTCTTTGTAACAACTTCCGT TGCTGCCATCGTAAACTGACACAGTGTTT	A_23_P1102	4.98	5.10	5.17	5.05	5.00	4.63	4.95	5.05
MYH3	NM_002470	CTAAGACTCGAGACTTCACCTCCAGCAGGAT GGTGGTCCACGAGAGTGAAGAGTGAGCCA	A_23_P26865	4.86	5.10	4.79	4.78	4.85	4.77	4.87	4.69

Supplementary Table 1 Transcripts mis-spliced in DM1 muscle tissue and other muscle transcripts that are expressed in human serum extracellular RNA (exRNA). Serum samples from healthy control subjects (Ctrl; N=7) were filtered through an 0.8 μm filter to remove cells, ultracentrifuged at 100,000 x g for 90 minutes to collect extracellular RNA in EVs and particles, and the EV/ribonucleoprotein (RNP) pellet was lysed. RNA was extracted using the Qiagen miRNeasy kit and examined by mRNA microarray analysis. The data are represented as quartile normalized with background subtraction and values indicate expression levels of each gene[10]. More than two-dozen transcripts of the mis-spliced mRNAs found in tibialis anterior (TA) muscle tissue of DM1 patients and reported as biomarkers of muscle weakness[2] are detected in the serum EV/RNP mRNA fraction and are candidate serum biomarkers. Note the similar levels of expression of muscle transcripts between healthy controls. The presence of muscle transcripts in EVs in this study is consistent with previous reports of EVs released by muscle cells[11] and measurement of muscle-enriched miRs in human serum[12].

Gene	Normalized	Raw
DMPK	2.348005036	380
INSR	5.410552617	2204
SOS1	11.78539296	4408
MBNL2	28.38510805	6004
CLASP1	10.58959612	3876
MBNL1	16.93241215	4180
MAP3K4	22.19040448	5472
NFIX	80.93189513	20292
NCOR2	17.48260768	6976
VPS39	16.7391543	3637
MAPT	3.968124064	1216
DMD	0.430326742	304
CNBP	92.40015841	14060
KIF13A	9.413147298	2736
CAPZB	184.636637	14197
CAMK2B	0.736559261	152
COPZ2	10.73770858	436
GFPT1	29.71567389	11546
IMPDH2	72.96428986	5548
ALPK3	1.393280124	684
ANK2	3.375451832	2204
ARFGAP2	30.19114602	3641
BIN1	13.30854948	1596
OPA1	15.35810568	4484
UBE2D3	53.16414472	6947
PHKA1	6.584110353	1824
PHKA2	3.110806445	698
LMNA	55.02644764	8284
TBC1D15	7.176337802	1900
FXR1	13.46978301	5396
ABLIM2	0.699642342	152
FHOD1	1.754220849	304
MLF1	1.303656519	152
DTNA	0.315128162	152
LDB3	0.25908561	76
TNNT2	1.443318118	76
TTN	0.166126138	836
MYH3	0.561759871	152
ACTA1	0	0
ATP2A1	0	0
CACNA1S	0	0
CKM	0	0
CLCN1	0	0
NRAP	0	0
PDLIM3	0	0
RYR1	0	0

Supplementary Table 2 Screening of human urine exosomes/microvesicles for presence of transcripts that are mis-spliced in DM1 muscle tissue. A previous study collected 3300 ml of urine from a human male, centrifuged the entire volume at low speed to pellet cells, passed the supernatant through a 0.8 μm filter to remove remaining debris, ultracentrifuged the filtered supernatant, isolated RNA from the exosome/microvesicle pellet using a commercially available kit (Qiagen), and identified genes present using massively parallel RNA sequencing[13]. Shown are raw and normalized counts of genes from that study that also were reported as biomarkers of DM1 in muscle biopsies[2], as well as other genes associated with muscular dystrophies. (Yellow: transcripts we report here that show differential alternative splicing in urine exRNA of DM1 individuals vs. DMD/BMD and UA controls).

Subjects	# Female	# Male	Total #	Mean age (years)	Median age (years)	Age range (years)
DM1	14	26	40	40	39	5 - 81
DM2	2	2	4	59	59	47 - 73
DMD/BMD	0	17	17	20	20	7 - 49
UA	22	7	29	n/a	n/a	n/a
Total	38	52	90	n/a	n/a	n/a

Supplementary Table 3 Subject enrollment. Of the forty subjects in the DM1 group, four were congenital, nine were childhood/juvenile onset, twenty-four were adult onset, and three were asymptomatic. For this study, we defined congenital onset DM1 using previously published criteria[14] that required both of the following: 1) DM symptoms beginning in the newborn period (< 30 days post-natal), including hypotonia, difficulty feeding, or respiratory difficulty that required hospitalization of more than 72 hours, and 2) genetic testing that identified a CTG repeat expansion in the *DMPK* gene in the child or mother. Childhood/juvenile onset was defined as DM symptoms beginning after the newborn period but before age 20 that led to diagnosis. Adult onset was defined as symptoms and diagnosis beginning age 20 and older. Asymptomatic individuals in the DM1 group were confirmed to have DM1 by genetic testing that was performed after one or more symptomatic family members were diagnosed by genetic testing.

Three of the four DM2 patients were diagnosed by genetic testing after an electromyogram (EMG) study unexpectedly found electrical myotonia, and the fourth DM2 subject was diagnosed by genetic testing after a symptomatic family member was diagnosed by genetic testing. None of the four DM2 subjects had grip myotonia evident on physical exam. Due to privacy considerations, we have no mean age data for the UA subjects. However, all were adults (> age 20 years), and either a spouse of a DM1 or DM2 participant, the parent of a DMD, BMD, or young DM1 participant. The mean age for the UA subjects is likely to be similar to, or perhaps slightly older, than the DM1 group.

Group	LE	Nitrates	Urobilinogen	Protein	pH	Blood	Specific gravity	Ketones	Bilirubin	Glucose
DM1	-	-	0.2	+/-	5	-	1.025	-	-	-
	-	-	0.2	+/-	6	-	1.020	-	-	-
	-	-	0.2	+/-	5	-	1.020	-	-	-
	-	-	0.2	+/-	5	-	1.030	-	-	-
	-	-	0.2	+/-	5	-	1.010	-	-	-
	-	-	0.2	+/-	5	-	1.020	-	-	-
	-	-	0.2	+/-	5	-	1.030	-	-	-
	+/-	-	0.2	-	6.5	-	1.005	-	-	-
	-	-	0.2	+/-	5	-	1.020	-	-	-
	-	-	0.2	-	5	-	1.030	-	-	-
	-	-	0.2	+/-	5	-	1.025	-	-	-
	-	-	0.2	+/-	5	-	1.025	-	-	-
	-	-	0.2	+/-	5	-	1.015	-	-	-
	-	-	1	+/-	6	-	1.010	-	-	-
	-	-	0.2	+/-	5	-	1.030	-	-	-
	-	-	0.2	-	6	-	1.005	-	-	-
	-	-	0.2	+/-	5	-	1.030	-	-	-
	-	-	0.2	+/-	6	-	1.015	-	-	-
	+/-	-	0.2	-	5	-	1.015	-	-	-
	70+	-	0.2	+/-	5	-	1.030	-	-	-
	-	-	0.2	-	5	-	1.030	-	-	-
	-	-	0.2	-	5	-	1.030	-	-	-
	-	-	0.2	-	6	-	1.020	-	-	-
	-	-	0.2	-	5	-	1.015	-	-	-
	-	-	0.2	-	5	-	1.015	-	-	-
	+/-	-	0.2	-	5	-	1.010	-	-	-
	+/-	-	0.2	-	6.5	-	1.015	-	-	-
	++	-	0.2	+/-	6.5	-	1.020	-	-	-
	-	-	0.2	-	6	-	1.010	-	-	-
	-	-	0.2	+/-	7.5	-	1.010	-	-	-
DMD/BMD	-	-	0.2	+/-	7.5	-	1.015	-	-	-
	-	-	0.2	+/-	6	-	1.015	-	-	-
	-	-	0.2	+/-	6	-	1.015	-	-	-
	-	-	0.2	+/-	7	-	1.010	-	-	-
	+/-	-	0.2	+/-	7	-	1.015	-	-	+
	+/-	-	0.2	-	6	-	1.025	-	-	-
	+/-	-	0.2	-	7	-	1.020	-	-	-
	-	-	0.2	-	6	-	1.030	-	-	-
	-	-	0.2	+/-	6.5	-	1.020	-	-	-
	-	-	0.2	+/-	6.5	-	1.015	-	-	-
	-	-	0.2	-	7.5	-	1.010	-	-	-
	-	-	0.2	-	7.5	-	1.010	-	-	-
	-	-	0.2	+/-	7	-	1.015	-	-	-
	-	-	0.2	-	5	-	1.020	-	-	-
UA	+	-	0.2	-	5	-	1.025	-	-	-
	-	-	0.2	-	6.5	-	1.010	-	-	-
	-	-	0.2	+/-	5	-	1.010	-	-	-
	-	-	0.2	+/-	6.5	-	1.010	-	-	-
	-	-	0.2	+/-	6	-	1.015	-	-	-
	-	-	0.2	+/-	5	-	1.015	-	1(17)+	-
	-	-	0.2	-	6.5	-	1.010	-	-	-
	-	-	0.2	+/-	5.5	-	1.010	-	-	-
	-	-	0.2	+/-	5	-	1.010	-	-	-
	-	-	0.2	+/-	6.5	-	1.010	-	-	-
	-	-	0.2	-	5	-	1.015	-	-	-
	-	-	0.2	+/-	7.5	-	1.010	-	-	-
	-	-	0.2	-	5	-	1.010	-	-	-
	+/-	-	0.2	-	5	-	1.015	-	-	-
	-	-	0.2	+/-	5	+/-	1.025	-	-	-
	+/-	-	0.2	-	6.5	+/-	1.010	-	-	-
	-	-	0.2	+/-	5	-	1.025	-	-	-
	-	-	0.2	+/-	7	-	1.010	-	-	-
	-	-	0.2	-	6	-	1.010	-	-	-
	-	-	0.2	+/-	5	+/-	1.030	-	-	-
	-	-	0.2	+/-	5	-	1.030	-	-	-
	-	8(140)	0.2	-	5	+++	1.020	40 (4.0) ++	-	1000(60)+++
	-	-	0.2	+/-	6	-	1.015	-	-	-
	-	-	0.2	-	6.5	-	1.005	-	-	-
	+/-	-	0.2	-	6	-	1.015	-	-	-

Supplementary Table 4 Clinical urinalysis data. We examined urine from a subset of 30 DM1, 14 DMD/BMD, and 25 UA subjects using urinalysis reagent strips (ACON Laboratories) as a general

screening tool for urinary tract disorders, endocrine disorders, or metabolic or systemic diseases that affect kidney function. LE = leukocyte esterase, a marker of white blood cells. Urobilinogen detection range is 0.2 - 1.0 mg/dL (3.5 - 200 μmol/L).

Transcript	Savkur, et al., 2001		Nakamori, et al., 2013				Current study	
	Exon inclusion % (range)		Exon inclusion %; mean ± SD (range)				Exon inclusion %	
	Various muscles (biopsy + autopsy)		Vastus lateralis (biopsy)		Tibialis anterior (biopsy)		Commercially available UA skeletal muscle RNA	
	DM1 (N = 17)	UA (N = 11)	DM1 (N = 16)	UA (N = 3)	DM1 (N = 50)	UA (N = 8)	RT-PCR	ddPCR
INSR exon 11	(5 - 42)	(52 - 78)	49 ± 18 (22 - 77)	~ 76 (70 - 78)	4 ± 4 (0 - 18)	69 ± 15 (50 - 100)	48	52
CLCN1 exon 7a	-	-	42 ± 16 (20 - 70)	~ 30 (25 - 35)	87 ± 9 (65 - 95)	31 ± 6 (25 - 35)	24	-
ATP2A1 exon 22	-	-	90 ± 9 (70 - 95)	99 (99)	32 ± 35 (0 - 80)	99 ± 0.1 (99)	100	-
MBNL2 exon 6	-	-	-	-	(10 - 45)	(2)	13	14
SOS1 exon 25	-	-	-	-	(5 - 40)	(95)	93	-
MBNL1 exon 7	-	-	-	-	(20 - 70)	(2 - 5)	19	27
CLASP1 exon 20	-	-	-	-	-	-	78	79
MAP3K4 exon 17	-	-	-	-	-	-	27	18
NFIX exon 7	-	-	-	-	(40 - 90)	(5)	54	-
NCOR2 exon 45a	-	-	-	-	(15 - 75)	(5 - 25)	25	-
VPS39 exon 3	-	-	-	-	(0 - 45)	(45 - 90)	35	-
MAPT exons 2, 3	-	-	-	-	-	-	96	-

Supplementary Table 5 Summary of alternative splicing patterns analyzed by RT-PCR in human skeletal muscle tissue from DM1 or UA subjects, as previously reported[2, 15]. The Savkur study examined splicing in a mixture of biopsy and autopsy specimens from various muscles. In the Nakamori study, vastus lateralis and tibialis anterior muscle biopsies from DM1 and UA subjects in non-overlapping cohorts were examined by RT-PCR, and a combination of muscle biopsy and autopsy specimens identified hundreds of candidate splicing defects in DM by microarray. Splicing of three transcripts, *INSR*, *CLCN1*, and *ATP2A1*, were analyzed in both the vastus lateralis and tibialis anterior biopsies, and exon inclusion percentages are displayed as mean ± SD, with the range of values from high to low shown in parentheses. The remaining values were adapted from scatter plot graphs and are displayed as a range. Note that the difference of exon inclusion between groups in the tibialis anterior was greater than the difference between groups in the vastus lateralis, which was attributed to the greater clinical involvement of tibialis anterior than vastus lateralis in DM1. Also note that, for most transcripts, exon inclusion showed a wider dynamic range in the DM1 group than the UA group. In the current study, we used commercially available total RNA isolated from skeletal muscle (which muscle is unknown) to measure splicing of *INSR*, *MBNL2*, *SOS1*, *MBNL1*, *CLASP1*, *MAP3K4*, *NFIX*, *NCOR2*, *VPS39*, and *MAPT* by RT-PCR, and *INSR*, *MBNL2*, *MBNL1*, *CLASP1*, *MAP3K4* by droplet digital PCR (ddPCR). For comparison, the RT-PCR alternative splicing patterns of *INSR*, *MBNL2*, *SOS1*, *MBNL1*, *CLASP1*, *MAP3K4*, *NFIX*, *NCOR2*, *VPS39*, and *MAPT* in urine exRNA are shown in Figs. 2 and 3, and the ddPCR alternative splicing patterns of *INSR*, *MBNL2*, *MBNL1*, *CLASP1*, and *MAP3K4* in urine exRNA are shown in Fig. 5. "-" = unexamined.

Transcript	PC1	PC2	PC3	PC4	PC5	PC6	PC7	PC8	PC9	PC10
INSR	-0.3256	-0.1724	0.0557	-0.2889	0.5294	-0.3167	0.1698	-0.5397	-0.2286	-0.1574
MBNL2	0.3420	-0.0510	-0.1895	0.0021	-0.2200	0.2735	0.0743	-0.7282	0.1003	0.4188
SOS1	-0.3410	-0.2187	0.1212	-0.0615	-0.0244	-0.3064	-0.2021	-0.0049	0.7519	0.3417
MBNL1	0.3349	-0.2454	-0.0386	-0.2761	-0.0792	0.0462	0.5019	0.0344	0.4562	-0.5295
CLASP1	-0.3342	0.0402	0.1947	0.2567	-0.4676	0.0869	-0.2944	-0.3849	0.0453	-0.5655
MAP3K4	0.3189	-0.0670	-0.3737	-0.3206	-0.2962	-0.5986	-0.4116	-0.0093	-0.1567	-0.1064
NFIX	0.2806	-0.4496	0.7281	-0.2557	-0.0684	0.1139	-0.2351	0.0374	-0.2021	0.0916
NCOR2	0.2911	-0.3628	0.0433	0.7754	0.1507	-0.3784	0.1024	-0.0481	-0.0226	-0.0362
VPS39	-0.2560	-0.6999	-0.4816	0.0036	0.0302	0.3834	-0.1791	0.1341	-0.1155	-0.0360
MAPT	-0.3257	-0.1729	0.0576	-0.0359	-0.5743	-0.2447	0.5648	0.0851	-0.2880	0.2460

Supplementary Table 6 The weights that were used to determine each principal component for calculation of the RT-PCR composite splicing biomarker scores shown in Fig. 4a. Note that for PC1, all ten transcripts contribute fairly evenly to the separation. "PC1" = first principal component, PC2 = second principal component, etc.

	INSR	MBNL2	MBNL1	CLASP1	MAP3K4	PC1	PC2
**PC1**	0.8794	-0.6092	-0.7601	0.7492	-0.8858	1	0.7575
**PC2**	0.9535	-0.0664	-0.2488	0.2084	-0.7562	0.7575	1

Supplementary Table 7 Principal component 1 (PC1) and principal component 2 (PC2) correlation coefficients for each transcript that was used to calculate the urine exRNA ddPCR-based composite biomarker scores shown in Fig. 9.

Gene	Left primer (5′ - 3′)	Right primer (5′ - 3′)	Target exon(s)	+ ex size (nt)	- ex size (nt)
ALPK3	GAGCTACCTGCTCAGCGTG	CTGTGACGATGCAGGTGAAC	2	194	155
ANK2	CCGATAACCAGCCTGAGACC	ACGGTGTGTCCATGCTCATC	21	223	130
ARFGAP2	GTGCCGTTCCTAATCACTCC	GTGAGGTGCCAAGCAGGTC	6	186	104
BIN1	AACCTCAATGATGTGCTGGTC	CTCTGGCTCGTGGTTGACTC	11	213	168
CACNA1S	TGATTGTCATTGGCAGCATC	AGGGTTCGCACTCCTTCTG	29	205	148
CAMK2B	AGCCATCCTCACCACCATG	AGGAGGAAGCGTCCCTTTG	13	217	142
CAPZB	AATCAGAAGTACGCTGAACGAG	CCTCCACCAGGTCATTCTTC	8	238	113
CLASP1	TATTGATGTGAACGCAGCAG	CCGGTTATCAGGTGTAGAGG	20	226	178
CLCN1	CCTGAAGGAATACCTCACAATG	TGAGGACAGCAGCACAGATG	7a	200	134
COPZ2	CGGCTTGACTGAACAGAGTG	CTGGCTGGAGACCTTAGGAG	9b	~320	213
DMD	TACGGTGACCACAAGGGAAC	TTTCACAGTGGTGCTGAGATAG	18 - 22 deletion	n/a	174
DMD	CCAGCTGGTTGAGCATTGTC	GTTCAGCTTCTGTTAGCCACTG	24 - 43 deletion	n/a	144
DMD	CAGTGGCTAACAGAAGCTGAAC	CGGTAATGAGTTCTTCCAACTG	45 - 50 deletion, ± 51 skipping	376	143
DMD	AATTGGGAAGCCTGAATCTG	CTCCGGTTCTGAAGGTGTTC	46 - 52 deletion	n/a	119
DMD	AGCCACTCAGCCAGTGAAG	GCAGAATAATCCCGGAGAAG	51 - 53 deletion	n/a	214
DMD	AGCCACTCAGCCAGTGAAG	CTCCGGTTCTGAAGGTGTTC	52 deletion, ± 51 skipping	416	183
DMD	GCTACCTGCCAGTGCAGAC	TGCGTGAATGAGTATCATCG	71	132	87
DMD	GAGCAACTCAACAACTCCTTCC	TAAGGACTCCATCGCTCTGC	78	128	96
FN1	CAAGGATGACAAGGAAAGTGTC	TGGACCAATGTTGGTGAATC	25	364	91
GFPT1	CCTCTGTTGATTGGTGTACGG	GTGCTGTCCACACGAGAGAG	9	170	116
IMPDH2	CCAGGCTGGTGTGGATGTAG	TTGTACACTGCTGTTGCTTGG	8 or 9	255	159
KIF13A	GGACACTGCCACTTATGGTTG	TGAGTGCATCTGACCACCTC	25	183	144
LMNA	AGATGACCTGCTCCATCACC	TACATGATGCTGCAGTTCTGG	11	321	171 (progerin)
MAP3K4	GGTACCTCGATGCCATAGTGAC	CAGCTATGGAAGCCAATCG	17	260	110
MAPT	CGAAGTGATGGAAGATCACG	GTGTCTCCAATGCCTGCTTC	2 + 3	309; 222	135
MAPT	AACGAAGATCGCCACACC	CCACTGCCACCTTCTTGG	10	296	242
MBNL1	CTGCCCAATACCAGGTCAAC	GGCTAGAGCCTGTTGGTATTG	7	230	176
MBNL2	CAGCACCAAGCCAACCAAG	GAGCCTGCTGGTAGTGCAAG	6	226	172
NCOR2	GCTGGAGGCCATAATTAGAAAG	GAGTGCACTGAGGAGACAGAG	45a	282	144
NFIX	AGCCCTGTTGATGACGTGTT	AGTGCAGGGCTGATGCTGT	7	250	127
NRAP	AGGCGCTGCACATTATCAC	ATAGCGGCCTCTCATGTGG	12	235	130
OPA1	GGATTGTGCCTGACATTGTG	CCACGATCTGTTGCTCTAAACG	4b	250	196
PHKA1	CTGGACCTGAGGGTAAGCTG	GGGAAGCCTGAAATAACTTCG	19	318	141
PHKA1	TATCCACGAGATTGGTGCTG	GTTGCCATTGACCTTGACG	28	212	173
SOS1	TGGTGCTTCCAGTACCACAG	GGCAGATTCTGGTCGTCTTC	25	208	163
UBE2D3	GTCGCCTGCTTTAACAATTTC	CTTGGGCAACTGTTCTCTTG	10	456	406
VPS39	TAGGATTCGGAAGGACGTTG	CTCACCGGTCTCTGTGTGC	3	274	241

Supplementary Table 8 PCR primers for human transcripts.

Gene	Left primer (5′ - 3′)	Right primer (5′ - 3′)	Target exon(s)	+ ex size (nt)	- ex size (nt)
Clasp1	GTCGACGACAGGATCTCTCC	GAGCTCTGCCGTCTCGTG	20	222	174
Map3k4	CAACAGAATCAGCGATGCCATC	TGGTCTGGCTGATGAGTGTTCG	17	355	199
Mapt	CCATGCTGGAGATTACACTCTG	GGCTTGGTCCTCCTGGTTC	2 + 3	283; 196	109
Mbnl1	ACCTGCAAGCCAAGATCAAG	TGTTGGCTAGAGCCTGTTGG	7	255	201
Ncor2	CAGGCGGTGCAAGAACAC	TTCGGCTGCTAGGTCTGC	45a	253	112
Nfix	TCGACGACAGTGAGATGGAG	CTGGATGATGGACGTGGAAG	7	293	170

Supplementary Table 9 PCR primers for mouse transcripts.

PP set	Left primer (5′ - 3′)	Right primer (5′ - 3′)	Probe (5′ - 3′)	Target exon	Amplicon size (nt)
CLASP1-20-21	GAGTACATAGGCCTGGACTCAG	CCGGTTATCAGGTGTAGAGG	AAAGCTCTGGGAGTGCCAC	+ 20	90
CLASP1-19-21	CATACGCATCCTTAGGTCGG	CCGGTTATCAGGTGTAGAGG	AAAGCTCTGGGAGTGCCAC	- 20	99
INSR-11-12	GCACAACGTGGTTTTCGTC	ACATCGCCAAGGGACCTG	AACCTCTTCAGGCACTGGTG	+ 11	93
INSR-10-12	TCCTCGTTTAGGAAGACGTTTG	ACATCGCCAAGGGACCTG	CCAGGCCATCTCGGAAAC	- 11	89
MBNL1-7-8	TCAGTCGGCTGTCAAATCAC	GAGCAGGCCTCTTTGGTAATG	TGACCTGGGAATTCCTCAAG	+ 7	98
MBNL1-6-8	CTGCCCAATACCAGGTCAAC	GAGCAGGCCTCTTTGGTAATG	CATGGGAATTCCTCAAGCTG	- 7	108
MBNL2-6-7	CTCGAAGCAACTGTAGACCTG	TTCAAGTGCTTGTCTCTTTGG	CCTGGTGCTCTTCATCCTTT	+ 6	72
MBNL2-5-7	CCACAGTCATGGCCTTTC	TTCAAGTGCTTGTCTCTTTGG	CCTGGTGCTCTTCATCCTTT	- 6	62
MAP3K4-17-18	ATCAGCAGTGCCCATGATAC	GACCTAAACTGCAATTCAGCAG	CCTGAAAATGATCGATTGGCTT	+ 17	86
MAP3K4-16-18	AGAGGGATTCAGGGGTTCC	GACCTAAACTGCAATTCAGCAG	CCTGAAAATGATCGATTGGCTT	- 17	75

Supplementary Table 10 ddPCR primer probe sets (PP sets) for human transcripts. All probes were 5′-labeled with Fam, and 3′-labeled with Black Hole Quencher-1 (BHQ-1) (Biosearch Technologies).

Transcript	*P* value	Are variances significantly different?	Variance greater in DM1 or UA?
**Urine exRNA ddPCR**			
DMPK copy number	0.1530	no	n/a
GTF2B copy number	< 0.0001	yes	DM1
DMPK vs. GTF2B	< 0.0001	yes	UA
**Urine cell total RNA ddPCR**			
DMPK copy number	0.1702	no	n/a
GTF2B copy number	0.8148	no	n/a
DMPK vs. GTF2B	0.7511	no	n/a
INSR exon 11 inclusion	0.0400	no	n/a
MBNL2 exon 6 inclusion	< 0.0001	yes	UA
MBNL1 exon 7 inclusion	0.0008	no	n/a
CLASP1 exon 20 inclusion	0.0159	yes	UA
MAP3K4 exon 17 inclusion	0.0136	no	n/a
**Serum exRNA ddPCR**			
DMPK copy number	0.5861	no	n/a
GTF2B copy number	0.7010	no	n/a
DMPK vs. GTF2B	0.0006	yes	UA
**Serum RT-PCR splicing**			
INSR ex 11	0.6479	no	n/a
MBNL2 ex 6	0.0787	no	n/a
SOS1 ex 25	0.9233	no	n/a
NFIX ex 7	0.1731	no	n/a
VPS39 ex 3	0.4998	no	n/a

Supplementary Table 11 Variance between groups. We used the F test to compare variances between DM1 and UA control groups for gene expression in urine and serum samples analyzed by ddPCR and splicing in serum samples by RT-PCR. In groups with statistically significant difference in variance, we used an unpaired two-tailed t-test with Welch's correction to determine differences between groups. The difference in variances of some groups may represent true differences in the two populations, and may be as important as the finding of different means (Figs. 1, 8, and Supplementary Fig. 6).

Supplementary Discussion

The potential to evaluate exRNA splicing outcomes as pharmacodynamic biomarkers in urine has the advantage of being non-invasive and can be repeated routinely over the course of treatment to evaluate drug target engagement. Our longitudinal monitoring of urine exRNA splicing patterns in DM1 and UA subjects over two years demonstrates feasibility and reliability of the assay. We also demonstrate that alternative splicing analysis by ddPCR using unique primer probe sets for each splice event offers the potential for greater quantitative accuracy of the larger exon inclusion products, and perhaps can increase throughput in a clinical trial or laboratory-based setting. Due to the need for general anesthesia and the absence of a therapeutic benefit, muscle biopsies generally are avoided in children with DM1. Consequently, detailed study of splicing outcomes in children with DM1 remains an unmet medical need. Urine exRNA should enable comprehensive non-invasive investigation of splicing outcomes in children with DM for the first time, facilitate clinical trials in these patients earlier, and perhaps enable convenient titration of dose. The shared pathogenic mechanism of alternative splicing misregulation in DM1 and myotonic dystrophy type 2 (DM2)[2] support our results that splicing in urine exRNA also may be useful for monitoring disease activity in DM2 patients.

Our finding that urine exRNA splicing outcomes of some transcripts demonstrate an early transition in asymptomatic patients, while other transcripts correlate with DM1 symptoms, is consistent with a previous study that found splicing of several transcripts in muscle biopsies showed a similar pattern[2]. Curiously, though, for three transcripts these properties appear to show the opposite pattern in urine exRNA as compared to tibialis anterior muscle biopsies in the previous study. For example, splicing of *INSR* in urine exRNA shows a wide dynamic range and appears related to symptom severity, but in tibialis anterior muscle biopsies showed a narrow dynamic range and an early transition in individuals with minimal symptoms. By contrast, splicing of *MBNL2* and *MBNL1* shows a narrow dynamic range and early transition in urine exRNA of asymptomatic individuals, but demonstrated a wide dynamic range and graded changes related to weakness of the tibialis anterior muscles biopsied in the previous study. This further argues against muscle tissue being the primary source of splice products found in urine exRNA. The reason that splicing outcomes are such powerful biomarkers of DM1 has to do with the disease mechanism involving mis-regulated alternative splicing[16], together with ratiometric measurements of exon inclusion/exclusion being inherently more sensitive than unidirectional changes that are typical of most biomarkers.

Exosomes and microparticles/microvesicles (MVs) are types of EVs distinguished by both size and origin. Exosomes (30 - 100 nm diameter) are released from multivesicular bodies when they fuse with the plasma membrane, while MVs (100 - 1000 nm diameter) form by exocytic budding[3]. The mean size of

EVs in our study suggests that the majority in urine were MVs, while most EVs in our serum samples were exosomes, suggesting the possibility that the mRNA splice products we are measuring are more prevalent in MVs than exosomes. Indeed, a recent study found that MVs contain a more accurate peripheral representation of glioblastoma cellular mRNA content than do exosomes[17]. Here we find that splice patterns of several transcripts in urine exRNA are more similar to those in kidney tissue and urothelial cells as compared to muscle tissue, suggesting that the exRNA found in urine may represent a pool from multiple different cell types along this urinary route. Our results showing ASO knockdown of *Dmpk* expression in mouse urinary tract tissues are consistent with earlier pre-clinical studies that demonstrated therapeutic ASO activity by inducing target knockdown and exon skipping in kidney tissue of mice and non-human primates[7, 18], and together suggest that ASOs could have similar effects in human kidney and other tissues lining the urinary tract that release exRNA into the urine.

Our finding that inclusion of *MAP3K4* exon 17 was different between males and females within both the DM1 and UA groups was unexpected, as was the significant correlation of exon inclusion with the CTG repeat length in DM1 males. The biological effect of a shift in the exon 17 inclusion percentage is unknown[19], although it is interesting to note that the sequence of this alternative exon includes ten CUG repeats, and that *Map3k4*-dependent signaling has been implicated in sex determination and testis development in mice[20].

In the clinical trial that led to accelerated approval of eteplirsen, immunofluorescence analysis of muscle biopsies demonstrated an increase in the number of muscle fibers expressing detectable dystrophin protein from a pre-treatment baseline of 1.1% to 17.4% after 180 weeks of treatment[21]. Exon skipping at the mRNA level was examined in these muscle biopsies using qualitative end-point RT-PCR and sequencing of PCR products; therefore, precise quantification of exon skipping activity in these biopsies is unavailable. In our study, it was surprising that exon 51 was mostly skipped in urine exRNA and urine cells of both individuals who are being treated with eteplirsen, and suggests that eteplirsen has greater uptake in cells and tissues lining the urinary tract than in deltoid or biceps muscle tissue, which were the two muscles that were examined in the clinical trials that led to accelerated approval of eteplirsen. This may be explained by pharmacokinetic properties of uncharged phosphorodiamidate morpholino ASOs like eteplirsen, which are excreted rapidly after systemic delivery, with little or negligible muscle tissue uptake[22].

The controversy surrounding the FDA accelerated approval of eteplirsen[21] indicates that realization of the full potential of exon skipping for DMD will require the development of new ASOs that outperform eteplirsen. For novel ASOs that engage their mRNA target in muscle and urinary tract tissues with similar efficacy, such as conjugated morpholinos[18] or ASOs that have phosphorothioate backbones[7]

(Supplementary Fig. 14), splicing measurements in urine RNA have the potential to correlate with drug activity in muscle tissue. Similarly, the absence of detectable pharmacological activity in urine RNA also could serve as an early indicator that the dose may be too low, or that the candidate drug eventually will fail, and thereby could help save valuable resources. These are important and potentially overlooked advantages of non-invasive monitoring of drug target engagement.

Collectively, our demonstration of disease-specific splice variants in urine exRNA suggest the value of biofluids as a means to identify novel splice variants that may help correlate genotype with phenotype for a number of diseases for which non-invasive biomarkers are unavailable. For example, in patients with Hutchinson-Gilford progeria syndrome (HGPS), point mutations in the *LMNA* gene activate a weak splice site in exon 11 that shortens the transcript and produces a truncated progerin protein[23]. ASOs that reduce use of this weak splice site are being evaluated as strategy to treat HGPS[24]. The presence of *LMNA* exon 11 in urine (Supplementary Fig. 2) suggests the potential use of exRNA to monitor drug effects in these patients as well. Our findings also support the study of exRNA from urine, serum, or CSF as a biomarker replacement for tissue biopsies in other diseases with altered mRNA splicing, including limb girdle muscular dystrophy type 1B, spinal muscular atrophy, and amyotrophic lateral sclerosis[25-27].

References

1. Krebs, S., Fischaleck, M. & Blum, H. A simple and loss-free method to remove TRIzol contaminations from minute RNA samples. *Anal Biochem* **387**, 136-138 (2009).
2. Nakamori, M. et al. Splicing biomarkers of disease severity in myotonic dystrophy. *Ann Neurol* **74**, 862-872 (2013).
3. Erdbrugger, U. & Le, T.H. Extracellular Vesicles in Renal Diseases: More than Novel Biomarkers? *J Am Soc Nephrol* **27**, 12-26 (2016).
4. Kanadia, R.N. et al. A muscleblind knockout model for myotonic dystrophy. *Science* **302**, 1978-1980 (2003).
5. Mankodi, A. et al. Myotonic dystrophy in transgenic mice expressing an expanded CUG repeat. *Science* **289**, 1769-1773 (2000).
6. Wheeler, T.M. et al. Targeting nuclear RNA for in vivo correction of myotonic dystrophy. *Nature* **488**, 111-115 (2012).
7. Pandey, S.K. et al. Identification and characterization of modified antisense oligonucleotides targeting DMPK in mice and nonhuman primates for the treatment of myotonic dystrophy type 1. *J Pharmacol Exp Ther* **355**, 329-340 (2015).
8. Bouge, A.L. et al. Targeted RNA-Seq profiling of splicing pattern in the DMD gene: exons are mostly constitutively spliced in human skeletal muscle. *Sci Rep* **7**, 39094 (2017).
9. Verheul, R.C., van Deutekom, J.C. & Datson, N.A. Digital Droplet PCR for the Absolute Quantification of Exon Skipping Induced by Antisense Oligonucleotides in (Pre-)Clinical Development for Duchenne Muscular Dystrophy. *PLoS One* **11**, e0162467 (2016).
10. Noerholm, M. et al. RNA expression patterns in serum microvesicles from patients with glioblastoma multiforme and controls. *BMC Cancer* **12**, 22 (2012).
11. Romancino, D.P. et al. Identification and characterization of the nano-sized vesicles released by muscle cells. *FEBS Lett* **587**, 1379-1384 (2013).
12. Aoi, W. et al. Muscle-enriched microRNA miR-486 decreases in circulation in response to exercise in young men. *Front Physiol* **4**, 80 (2013).
13. Miranda, K.C. et al. Massively parallel sequencing of human urinary exosome/microvesicle RNA reveals a predominance of non-coding RNA. *PLoS One* **9**, e96094 (2014).
14. Campbell, C., Levin, S., Siu, V.M., Venance, S. & Jacob, P. Congenital myotonic dystrophy: Canadian population-based surveillance study. *J Pediatr* **163**, 120-125 e121-123 (2013).
15. Savkur, R.S., Philips, A.V. & Cooper, T.A. Aberrant regulation of insulin receptor alternative splicing is associated with insulin resistance in myotonic dystrophy. *Nat Genet* **29**, 40-47 (2001).
16. Lin, X. et al. Failure of MBNL1-dependent post-natal splicing transitions in myotonic dystrophy. *Hum Mol Genet* **15**, 2087-2097 (2006).

17. Wei, Z. et al. Coding and noncoding landscape of extracellular RNA released by human glioma stem cells. *Nat Commun* **8**, 1145 (2017).
18. Morcos, P.A., Li, Y. & Jiang, S. Vivo-Morpholinos: a non-peptide transporter delivers Morpholinos into a wide array of mouse tissues. *Biotechniques* **45**, 613-614, 616, 618 passim (2008).
19. Gonzalez-Barriga, A. et al. Design and analysis of effects of triplet repeat oligonucleotides in cell models for myotonic dystrophy. *Mol Ther Nucleic Acids* **2**, e81 (2013).
20. Bogani, D. et al. Loss of mitogen-activated protein kinase kinase kinase 4 (MAP3K4) reveals a requirement for MAPK signalling in mouse sex determination. *PLoS biology* **7**, e1000196 (2009).
21. Aartsma-Rus, A. & Krieg, A.M. FDA Approves Eteplirsen for Duchenne Muscular Dystrophy: The Next Chapter in the Eteplirsen Saga. *Nucleic Acid Ther* **27**, 1-3 (2017).
22. Bennett, C.F., Baker, B.F., Pham, N., Swayze, E. & Geary, R.S. Pharmacology of Antisense Drugs. *Annu Rev Pharmacol Toxicol* **57**, 81-105 (2017).
23. Eriksson, M. et al. Recurrent de novo point mutations in lamin A cause Hutchinson-Gilford progeria syndrome. *Nature* **423**, 293-298 (2003).
24. Lee, J.M. et al. Modulation of LMNA splicing as a strategy to treat prelamin A diseases. *J Clin Invest* **126**, 1592-1602 (2016).
25. Hua, Y. et al. Peripheral SMN restoration is essential for long-term rescue of a severe spinal muscular atrophy mouse model. *Nature* **478**, 123-126 (2011).
26. Honda, D. et al. The ALS/FTLD-related RNA-binding proteins TDP-43 and FUS have common downstream RNA targets in cortical neurons. *FEBS Open Bio* **4**, 1-10 (2013).
27. Genschel, J. & Schmidt, H.H. Mutations in the LMNA gene encoding lamin A/C. *Hum Mutat* **16**, 451-459 (2000).
